# Taxonomic and functional metagenomic assessment of a *Dolichospermum* bloom in a large and deep lake south of the Alps

**DOI:** 10.1093/femsec/fiae117

**Published:** 2024-09-03

**Authors:** Nico Salmaso, Leonardo Cerasino, Massimo Pindo, Adriano Boscaini

**Affiliations:** Research and Innovation Centre, Fondazione Edmund Mach, Via Edmund Mach, 1, 38098 San Michele all'Adige, Italy; NBFC, National Biodiversity Future Center, Palermo 90133, Italy; Research and Innovation Centre, Fondazione Edmund Mach, Via Edmund Mach, 1, 38098 San Michele all'Adige, Italy; Research and Innovation Centre, Fondazione Edmund Mach, Via Edmund Mach, 1, 38098 San Michele all'Adige, Italy; Research and Innovation Centre, Fondazione Edmund Mach, Via Edmund Mach, 1, 38098 San Michele all'Adige, Italy

**Keywords:** ADA *Anabaena/Dolichospermum/Aphanizomenon*, cyanobacterial blooms, *Dolichospermum*, genome mining, KEGG functional analysis, metagenome assembled genome

## Abstract

Untargeted genetic approaches can be used to explore the high metabolic versatility of cyanobacteria. In this context, a comprehensive metagenomic shotgun analysis was performed on a population of *Dolichospermum lemmermannii* collected during a surface bloom in Lake Garda in the summer of 2020. Using a phylogenomic approach, the almost complete metagenome-assembled genome obtained from the analysis allowed to clarify the taxonomic position of the species within the genus *Dolichospermum* and contributed to frame the taxonomy of this genus within the ADA group (*Anabaena*/*Dolichospermum*/*Aphanizomenon*). In addition to common functional traits represented in the central metabolism of photosynthetic cyanobacteria, the genome annotation uncovered some distinctive and adaptive traits that helped define the factors that promote and maintain bloom-forming heterocytous nitrogen-fixing Nostocales in oligotrophic lakes. In addition, genetic clusters were identified that potentially encode several secondary metabolites that were previously unknown in the populations evolving in the southern Alpine Lake district. These included geosmin, anabaenopetins, and other bioactive compounds. The results expanded the knowledge of the distinctive competitive traits that drive algal blooms and provided guidance for more targeted analyses of cyanobacterial metabolites with implications for human health and water resource use.

## Teaser

The metagenomic analysis of *Dolichospermum* collected during a bloom allowed to clarify its taxonomic position within the *Anabaena*/*Dolichospermum*/*Aphanizomenon* (ADA) classification scheme, and to characterize the distinctive competitive traits that drive oligotrophic algal blooms.

## Introduction

Cyanobacteria are a group of photosynthetic prokaryotic microorganisms that are widely distributed throughout the world. In aquatic environments, cyanobacteria are essential for the sustainability of terrestrial life, accounting for ~25% of carbon dioxide fixation (Aguiló-Nicolau et al. 2023). In phosphorus- and nitrogen-rich lake and river ecosystems, this group of microorganisms is often able to reproduce very rapidly, producing high biomasses and causing blooms (Reynolds and Walsby 1975). In addition to eutrophication, cyanobacterial blooms are favoured and intensified by high water temperatures and thermal stability of the water column (Paerl and Huisman 2009, Visser et al. 2016, Jankowiak et al. 2019). Evidence of an increase in the frequency, size and duration of cyanobacterial blooms around the world has been reported (Huisman et al. 2018, Hou et al. 2022). These phenomena are influenced by geographic location, lake and watershed characteristics and the species involved, and their documentation depends on monitoring coverage and effort (Wood et al. 2017, Hallegraeff et al. 2021, Bullerjahn et al. 2023, Mishra et al. 2023, Erratt and Freeman 2024). Given that many cyanobacteria are capable of producing a wide range of secondary metabolites that are toxic to humans and animals (Meriluoto et al. 2017), cyanobacterial blooms require special attention in terms of monitoring and risk assessment related to the use of aquatic resources for drinking and bathing purposes (Chorus and Welker 2021).

The dynamics of cyanobacterial harmful algal blooms can be highly variable, ranging from localized and episodic events over a few hours or days to persistent, large biomass accumulations over large areas for several days or weeks (Stumpf et al. 2012, Steffen et al. 2017). The intensity of these blooms depends on nutrient availability and local climatic and hydrological conditions (Wynne et al. 2010, Wu et al. 2013).

The environmental localization and impact of cyanobacterial blooms are highly species-specific, depending on the vertical accumulation of biomass, e.g. at the surface, dispersed in the water column, or forming metalimnetic blooms, as in the case of *Planktothrix rubescens* (De Candolle ex Gomont) Anagnostidis and Komárek (Lindholm et al. 1989, Codd et al. 1999, Boscaini et al. 2017, Zepernick et al. 2024). In turn, the ability to synthesize toxins is often strain-specific and characterized by strong geographic patterns (Kardinaal et al. 2007, Haande et al. 2008, Vico et al. 2020). In all these cases, a complete taxonomic and functional characterization of the events is essential for a comprehensive risk assessment and management of the affected waters.

The conventional taxonomic approach involves the use of microscopic observations of environmental samples, occasionally coupled with genetic characterization of isolates and/or environmental samples (Kurmayer et al. 2017). In parallel, a range of different cyanotoxins is characterized and quantified using liquid chromatography-mass spectrometry (LC-MS) or enzyme-linked immunosorbent assay (Meriluoto et al. 2017). Overall, the genetic analysis of isolates and the metabolomic profiling of isolates and environmental samples are based on targeted analyses, which remain an efficient approach to ensure the correct identification of cyanotoxin producers. However, the use of targeted analyses is often very demanding, requiring fully equipped laboratories and, in the case of isolates, long periods of time required for the establishment and growth of populations. They are also restricted to a limited number of target genes and metabolites.

More recently, conventional approaches have been complemented by a number of technologies using culture-independent high-throughput sequencing (HTS) approaches (Thompson and Thielen 2023). Metabarcoding has been widely used as a fast and inexpensive tool to characterize the microbial and cyanobacterial communities (Pawlowski et al. 2018, Cordier et al. 2020, Domaizon et al. 2021), allowing the study of spatial and temporal patterns in the distribution of specific cyanobacterial oligotypes (Berry et al. 2017, Salmaso et al. 2024) and toxigenic taxa (Casero et al. 2019, Linz et al. 2023). Analogous to the classical approaches, metabarcoding is based on the targeted analysis of short DNA (deoxyribonucleic acid) amplicons, allowing a deep determination of microbial communities, but with many limitations, mainly due to the use of single marker genes per run, the limited information carried by short DNA fragments, and the incompleteness of reference databases (Malashenkov et al. 2021, Salmaso et al. 2022). Conversely, metagenomic approaches are based on DNA-targeted independent methods that allow the reconstruction of metagenome-assembled genomes (MAGs) from the analysis of any type of biological and environmental samples (Quince et al. 2017, Pérez-Cobas et al. 2020). The use of draft genomes, i.e. MAGs reconstructed with different levels of completeness and contamination (Garner et al. 2023), allows to unravel the taxonomy and phylogeny of microbial assemblages (Soo et al. 2014, Dvořák et al. 2023, Pessi et al. 2023, Strunecký et al. 2023), which opens important perspectives for the determination of functional properties of species and communities (Chrismas et al. 2018, Linz et al. 2018, Alcorta et al. 2020, Tran et al. 2021, Van Le et al. 2024).

In this work, we report the results of a full-shotgun metagenomic analysis performed on a sample collected during a summer bloom of *Dolichospermum* detected in Lake Garda. In this context, and considering the many definitions proposed (Zepernick et al. 2024), the term bloom is applied to indicate a visible formation of scum. Blooms with the same characteristics have been recorded irregularly since the early 1990s, and the taxonomy of the unique species involved has been characterized (Salmaso et al. 2015b, Capelli et al. 2017). Our main objectives were (i) to use the MAG of *Dolichospermum* to characterize the taxonomic assignment of the species at the genomic level; (ii) to identify, through genome annotation, the main metabolic pathways and the presence of relevant metabolites in *Dolichospermum*, including cyanotoxins; and (iii) to discuss the prospects for the practical use of metagenomic approaches to complement conventional monitoring in assessing the risks posed by the development of potentially toxigenic cyanobacterial populations.

## Materials and methods

### Sampling, filtration, and phytoplankton analysis

The sample for metagenomic and cyanotoxins analyses was collected on the surface using a sterilized plastic bottle during a bloom observed on the afternoon of September 1, 2020, in the shallower southeastern basin of Lake Garda, ~3 km off the coast of the village of Bardolino (45.55 N 10.68 E; Fig. S1). The sampled layer ranged from 2 to 10 cm. The sample was kept refrigerated overnight until filtration, which was performed the next day on GF/C filters (nominal particle retention 1.2 µm) until almost clogged.

During the bloom, water temperatures were measured with multiparameter probes (Idronaut Ocean Seven 316 Plus and SBE 19plus SeaCAT). Water transparency was measured with a Secchi disk. Samples for chemical (0–2, 9–11, and 19–21 m) and phytoplankton (0–20 m) analyses were collected by the Regional Agency for Environmental Protection and Prevention of the Veneto Region (ARPAV) (Ragusa et al. 2021). The used methods have been regularly checked between the ARPAV and the Fondazione Mach of S. Michele all'Adige (FEM) laboratory as part of the activities carried out within the Long Term Ecological Research (LTER) network (Capotondi et al. 2021) and previous projects (Domaizon et al. 2021). Chemical analyses were performed according to standard methods (APHA, AWWA, and WEF 2018) and included pH, dissolved oxygen, sulfate (SO_4_^2−^), nitrogen (NO_3_-N, NH_4_-N and TN, total nitrogen) and phosphorus (SRP, soluble reactive phosphorus and TP, total phosphorus). Phytoplankton analyses were performed using inverted microscopes (Salmaso et al. 2022). On the same day, additional field measurements and samples for chemical and phytoplankton analyses were collected in the deeper northwestern basin (45.69 N, 10.72 E), ~20 km north of the bloom location.

### Cyanotoxins analyses

Cyanotoxins were extracted from a GF/C filter and quantified as described by Cerasino et al. (2017) and Cerasino and Salmaso (2020). The extraction was performed in acetonitrile/water (60/40 v/v), containing 0.1% formic acid. Extracts were analysed using an LC-MS/MS system, composed of a Waters Acquity UPLC system (Waters, Milford, MA, USA) coupled to a SCIEX 4000 QTRAP mass spectrometer (AB Sciex Pte. Ltd., Singapore). The most common microcystins (MCs) structural variants were quantified, including MC-RR, MC-[D-Asp3]-RR (RRdm), MC-[D-Asp3]-HtyrR (HtyRdm), MC-YR, MC-LR, MC-[D-Asp3]-LR (LRdm), MC-WR, MC-LA, MC-LY, MC-LW, MC-LF (limit of detection, LOD, 0.5–9 ng/g d.w.) (Cerasino and Salmaso 2020). Details on the analyses of anatoxins (ATXs) (ATX-a and homoATX-a; LOD, 2.0–4.0 ng/g d.w.), cylindrospermopsin (CYN; LOD, 0.4 ng/g d.w.), and saxitoxins (SXTs) (STX, dcSTX, NeoSTX, GTX1, GTX4, GTX5, C1, and C2; LOD 5.0–27.0 ng/g d.w) were reported in (Ballot et al. 2020).

### DNA extraction, library preparation and sequencing

Filters were stored at –20°C until DNA extraction, which was performed with DNeasy PowerWater® DNA Isolation Kit (Qiagen, USA). DNA concentrations were measured with a NanoDrop ND-8000 (Thermo Fisher Scientific Inc., USA). Starting from a total amount of 100 ng, total DNA was fragmented by enzymatic reaction at 37°C x 5 min producing fragments of 500 bp. Paired-end library was prepared using the KAPA HyperPlus kit (Roche). Adapters from the KAPA Unique Dual-Indexed Adapter Kit (Roche) recommended for use with the KAPA HyperPlus Kit were ligated to the DNA fragments following the manufacturer's instructions. Libraries were quantified using the KAPA Library Quantification Kits (Roche) and were sequenced for 150 bp paired-end reads on the Illumina Novaseq-6000 platform (Illumina Inc., San Diego, CA, USA).

### Assembling and binning

Paired raw reads were checked with FastQC 0.12.1 (github.com/s-andrews/FastQC). Removal of residual adapters and PhiX contaminated reads, and trimming (trimq = 18, maq = 20, maxns = 0, minlen = 35) were performed using BBDuk (BBMap version 39.05; https://jgi.doe.gov). Human DNA reads were mapped using Bowtie 2.5.2 (Langmead and Salzberg 2012) against the corresponding human reference genome, GRCh38.p14 (GCF_000001405.40) and filtered with SAMtools 1.19 (Danecek et al. 2021). For de Bruijn graph assemblers, a very high coverage depth amplifies the effect of errors on the assembly graph and may even confuse error correction algorithms (Lapidus and Korobeynikov 2021). To cope with the high coverage of reads characterizing *Dolichospermum* (1015× with relative abundance 29.5% after assembly based on the entire set of reads), the paired-end reads were therefore subsetted using BBMap reformat.sh, at a samplerate = 0.3. An assessment of the taxonomic composition of the microbial community with species-level resolution using the filtered reads was carried out using MetaPhlAn 4.0.6 with the –unclassified_estimation parameter (Blanco-Miguez et al. 2022), and results converted from NCBI (National Center for Biotechnology Information) to GTDB (Genome Taxonomy Database) taxonomy with the MetaPhlan utility script sgb_to_gtdb_profile.py.

Filtered reads were corrected with metaSPAdes 3.15.5 (Nurk et al. 2017) (–only-error-correction) and thereafter assembled with Megahit 1.2.9 (Li et al. 2015) (–presets meta-sensitive). After discarding contigs shorter than 1000 bp and simplifying the contigs names with Anvi'o 8 (Eren et al. 2021), the contigs were binned using CONCOCT 1.1.0 (Alneberg et al. 2014), MetaBAT 2.17–21 (Kang et al. 2019), and SemiBin2 2.0.2 (Pan et al. 2022), and results combined using DAS Tool 1.1.6 (Sieber et al. 2018), using default options and score threshold = 0.4. The resulting MAGs were assessed for the presence of chimerism using GUNC 1.0.6 (Orakov et al. 2021). Additional sources of bacterial and eukaryotic contamination were checked using MDMcleaner 0.8.7 (Vollmers et al. 2022) and Whokaryote 1.1.2 (Pronk and Medema 2022), and results were assessed manually. The *Dolichospermum* bin (FEM_B0920) was further checked and confirmed with Anvi'o 8 (Eren et al. 2021). Completeness and redundancy (Bowers et al. 2017) of MAGs were estimated using CheckM 1.2.2 (Parks et al. 2015) and CheckM2 1.0.2 (Chklovski et al. 2023). Coverage of the individual MAGs was computed using CoverM 0.6.1 (github.com/wwood/CoverM).

The Whole Genome Shotgun project has been deposited at DDBJ/ENA/GenBank under the project number PRJNA1074715.

### Taxonomic assignment and phylogenomic analyses

The taxonomic analysis of the MAGs recovered from the Lake Garda bloom was based on the Genome Taxonomy Database (GTDB) 09-RS220, released in April 2024 (Parks et al. 2022). Taxonomic classifications were performed using GTDB-Tk 2.4.0 updated to use the GTDB R220 taxonomy (Chaumeil et al. 2022).

Genomes to be compared with the *Dolichospermum* MAG determined in Lake Garda (FEM_B0920) were selected to cover all the *Dolichospermum* species available in GTDB R220. Most of these genomes were obtained from metagenomic analyses of non-axenic cultures, enriched cultures, and environmental samples. Only in a few cases, DNA was isolated from single cells (*Dolichospermum* spp., strains sed1-sed10; Woodhouse et al. 2024). In the GTDB R220 taxonomy no *Dolichospermum lemmermannii* (Richter) P. Wacklin, L. Hoffmann, and J. Komárek genomes were included, whereas in NCBI (Sayers et al. 2022), two genomes attributable to this species were reported. The first was *D. lemmermannii* CS-548, collected in 1981 from Lake Edlandsvatnet, Norway (GCA_028330815.1) and classified in the GTDB R220 under *Dolichospermum* sp000312705. The second was *Dolichospermum* SB001 (GCA_016462165.1), which was detected during an offshore bloom of *D. lemmermannii* in Lake Superior in August 2018 (Sheik et al. 2022); in GTDB R220, this genome was however not included in the reference database. From this initial set, three genomes lacking NCBI genus-level classification and GTDB species classifications, and a further 12 genomes with completeness below 95% and/or contamination above 4% (as determined by CheckM2) were excluded from subsequent analyses. Similarly, *Dolichospermum* SB001 (87.8% completeness and 0.2% contamination) was not included in the main set of analyses. The genomes analysed are reported in Table S1.

The MAG of *Dolichospermum* recorded in Lake Garda (GCA_037075685.1) was compared with this set of genomes using the Average Nucleotide Identity (ANI) (Palmer et al. 2020) computed using pyani 0.2.12 (ANI_b_) (Pritchard et al. 2015), OrthoANIu 1.2 (Yoon et al. 2017), and fastANI 1.32 (Jain et al. 2018). The suggested species boundary for distinguishing between two species based on ANI values is 0.95–0.96 (Goris et al. 2007, Richter and Rosselló-Móra 2009), whereas genomes of different species generally have ANI < 0.90 and ANI values in the range 0.90–0.95 are comparatively rare (Rodriguez-R et al. 2024).

Phylogenomic analyses were carried out using GToTree 1.8.6 (Lee 2019) with the parameter -G set to 0.75. GToTree makes use of Muscle 5 (Edgar 2022) to align sequences. Sequence alignments were computed using the pre-packaged HMM single-copy genes set specific for Cyanobacteria (251 genes) available in GToTree. The alignment and partitions obtained with GToTree were used to build phylogenomic trees with IQ-TREE 2.3.4, using ModelFinder to select the substitution mode (Nguyen et al. 2015), and with branch supports computed using ultrafast bootstrap (UFBoot) values (Minh et al. 2013) with 10 000 replicates; UFBoot 95% support values roughly correspond to a probability of 95% that a clade is true. Two phylogenomic analyses were performed, the first including only the genomes classified at the species level in the GTDB R220 taxonomy (58 genomes) and the second including all the available *Dolichospermum* genomes (96 genomes); besides the *Dolichospermum* collected in Lake Garda, in both cases, the genome of *Cuspidothrix issatschenkoi* CHARLIE-1 (GCF_002934005.1) was used as an outgroup, resulting in a total of 60 and 98 genomes being utilized in the respective analyses. The trees were built using iTOL v6 (Letunic and Bork 2024). Analyses were performed by calculating the alignment and trees using both protein and DNA sequences (Lee 2019), which yielded comparable results; only the trees constructed using proteins are shown. Besides the GTDB taxonomy, the clades obtained in the trees were interpreted taking into account the NCBI taxonomy and the classifications based on the ADA (*Anabaena*/*Dolichospermum*/*Aphanizomenon*) clade concept (Driscoll et al. 2018, Dreher et al. 2021).

### Functional annotation

Functional annotation of the *Dolichospermum* draft genome was performed using the NCBI stand-alone software package Prokaryotic Genome Annotation Pipeline (PGAP) 2023–10–03.build7061 (github.com/ncbi/pgap) (Li et al. 2020) and finally confirmed by annotation using the PGAP service in NCBI (https://www.ncbi.nlm.nih.gov/). PGAP allows the prediction of protein-coding genes and other functional genomic entities such as structural RNAs, tRNAs, small RNAs and pseudogenes. Functional annotations have been integrated with Bakta 1.9.2, which assigns stable database identifiers from RefSeq and UniProt (Schwengers et al. 2021) and, to improve the annotation of antimicrobial resistance genes (ARGs), AMRFinderPlus (Feldgarden et al. 2019, Schwengers et al. 2021). Antimicrobial resistance (AMR) and ARGs were further predicted using ABRicate (version 1.0.1), incorporating the NCBI AMRFinder, ARG-ANNOT, ResFinder, and Card databases (github.com/tseemann/abricate) with minimum DNA identity and coverage values of 80% and 50%, respectively. The location of ribosomal rRNA genes in MAGs was further evaluated using Barrnap 0.9 (github.com/tseemann/barrnap).

Basic metabolism and phenotypic features of the NCBI *D. lemmermannii* species were defined using the Kyoto Encyclopedia of Genes and Genomes (KEGG) (Kanehisa et al. 2014). After identifying proteins with Prodigal 2.6.3 (Hyatt et al. 2010), the functional orthologs defined by K numbers (Kegg Orthology, KO, identifiers) were determined using GhostKOALA computed with the genus_prokaryotes + viruses database file (Kanehisa et al. 2016). The pathway KEGG modules (functional units of gene sets in metabolic pathways) were identified with the KEGG Mapper Reconstruct tool (Kanehisa and Sato 2020). Selected phenotypic traits were analysed using KEGG pathway maps (Kanehisa et al. 2022).

The presence of secondary metabolite biosynthetic gene clusters (BGCs) in the *Dolichospermum* genomes was assessed using the antibiotics and secondary metabolite analysis shell antismash 7.1.0 (default mode), which allows the detection and characterization of BGCs in microorganisms. The similarity is defined as the percentage of genes within the closest known compound that have a significant BLAST hit to genes within the current region (Blin et al. 2023).

Identification of target genes encoding cyanotoxins (Kurmayer et al. 2017) and geosmin (GEO) (Suurnäkki et al. 2015) in the *Dolichospermum* genomes and contigs was performed using specific gene databases with ISeqDb 0.0.3 (github.com/hts-tools/iseqdb). Selected markers included *anaC* and *anaF* (anatoxin-a, ATX-a), *mcyB, mcyD* and *mcyE* (microcystins), *cyrJ* (cylindrospermopsin), *sxtA* (saxitoxin) and *geoA* (geosmin) downloaded from GenBank and included in the ISeqDb package.

## Results

### The *Dolichospermum* bloom

Shortly after sampling at the LTER station in the northeastern basin, an opportunistic sample was taken on the afternoon of September 1, 2020, from a bloom ~3 km off the coast of the village of Bardolino. The bloom was observed during a period of calm winds. The bloom had the same characteristics observed in other episodes documented in previous years (Salmaso et al. 2015b), i.e. with distinct aggregates of filaments visible with the naked eye and more or less dense streaks in the first few cm of the water column (Fig. S2).

### Cyanotoxins

The LC-MS analyses showed a quantifiable presence of ATX-a (0.3 µg L^−1^). The other toxins analysed were not detected.

### Environmental and light microscopy analyses

During the bloom, water temperatures in the first 20 m were between 21.0 and 23.6°C (Table 1A). The Secchi disk depth was 4 m. pH and oxygen values were between 8.1 and 8.5, and 7.7 and 9.4 mg L^−1^ (87%–120% saturation), respectively. Sulfate showed homogeneous concentrations in the layers analysed (10 mg L^−1^). SRP and TP were extremely low throughout the epilimnion, below the detection limit and < 10 µg L^−1^, respectively. In the first 10 m, nitrate nitrogen was at or below the detection limit (0.05 mg L^−1^). In the northwestern basin, the analyses gave comparable results, with the main difference being the more homogeneous and measurable concentrations of NO_3_-N (120–190 µg L^−1^) and dissolved oxygen, and slightly higher SRP and TP concentrations in the epilimnion (Table 1B).

**Table 1.** Physical and chemical characteristics of samples collected in three discrete epilimnetic layers at the (A) southeastern and (B) northwestern stations of Lake Garda during the *Dolichospermum* bloom recorded in the southeastern basin.

	(A)			(B)		
	South East station (Bardolino)			North West station (Brenzone)		
Variable/water layer	0–2 m	9–11 m	19–21 m	0–2 m	9–11 m	19–21 m
Temperature (°C)	23.6	23.4	21.0	22.5	21.6	20.5
pH	8.4	8.5	8.1	8.6	8.6	8.5
Oxygen (mg L^−1^)	9.4	9.3	7.7	10.0	9.7	9.5
Sulfate, SO_4_^2−^ (mg L^−1^)	10	10	10	9	9	10
NO_3_-N (mg L^−1^)	< 0.05	< 0.05	0.14	0.164	0.185	0.121
NH_4_-N (mg L^−1^)	< 0.01	< 0.01	< 0.01	0.012	0.006	0.008
TN (mg L^−1^)	< 0.5	< 0.5	< 0.5	0.25	0.22	0.24
SRP (µg L^−1^)	< 5	< 5	< 5	< 5	< 5	< 5
TP (µg L^−1^)	5	8	7	12	11	11
Secchi disk depth (m)	4			7		

The microscopic analyses performed by ARPAV confirmed the presence of *D. lemmermannii* in the integrated sample collected between 0 and 20 m. The total biovolume contributed by the whole phytoplankton community was ~400 mm^3^ m^−3^, while the contribution of cyanobacteria was ~100 mm^3^ m^−3^. More than 60% of the biovolume of cyanobacteria was contributed by picoplankton, filaments of *Planktothrix rubescens* (De Candolle ex Gomont) Anagnostidis and Komárek and colonies of *Microcystis aeruginosa* (Kützing) Kützing, while the contribution of *D. lemmermannii* was much lower, i.e. around 8% (< 10 mm^3^ m^−3^). *Tychonema bourrellyi* (J.W.G. Lund) Anagnostidis and Komárek was detected with a fraction of biovolume around 5%. Eukaryotic phytoplankton was mainly dominated by Chlorophyceae (142 mm^3^ m^−3^; *Hariotina reticulata* (P.A. Dangeard) and *Monactinus simplex* (Meyen) Corda); Bacillariophyceae (60 mm^3^ m^−3^; *Fragilaria crotonensis* Kitton); Dinophyceae (36 mm^3^ m^−3^; *Ceratium hirundinella* (O.F. Müller) Dujardin); Trebouxiophyceae (27 mm^3^ m^−3^; *Mucidosphaerium pulchellum* (H.C. Wood) C. Bock, Proschold, and Krienitz); and Cryptophyceae (21 mm^3^ m^−3^; *Plagioselmis nannoplanctica* (Skuja) G. Novarino, I.A.N. Lucas, and Morrall). No *Dolichospermum* blooms were visually detected at the north-western LTER station. In the 0–20 m layer of this station, the average total cyanobacterial biovolume was 106 mm^3^ m^−3^, of which 2 mm^3^ m^−3^ were contributed by *D. lemmermannii* (Fig. S3).

### Metagenomes assembly and the *Dolichospermum* MAG

Novaseq sequencing generated 62 967 310 paired-end reads. In total, raw data quality processing removed around 11% of the raw reads. After resampling, the successive analyses were performed on the 30% of the quality checked and processed paired-end reads. After correction with metaSPAdes, assembly with Megahit yielded 16 508 contigs larger than 1000 bp, with a total size of 76 Mbp and N50 of 18 548 bp.

The *Dolichospermum* FEM_B0920 was 4.8 Mbp assembled into 189 contigs, with N50 40 920 bp, GC content 38.1%, and coverage 305×; based on CheckM2, completeness and contamination estimates were 99.9% and 0%, respectively (Table 2).

**Table 2.** Summary of statistics from the *Dolichospermum lemmermannii* FEM_B0920 genome assembly.

Variable	
Total length (bp)	4 787 045
No. of contigs	189
GC content (%)	38.13
Mean coverage (×)	305
Size of longest contig (bp)	115 416
N50 (bp)	40 920
No. of protein-coding genes	4439
No. of tRNA	40
Complete 5S rRNA	3
Complete 16S rRNA	1
Complete 23S rRNA	1
Completeness (CheckM) (%)	99.67
Contamination (CheckM) (%)	0.22
Strain heterogeneity (CheckM) (%)	0.0
Completeness (CheckM 2) (%)	99.94
Contamination (CheckM 2) (%)	0.0

According to the GTDB taxonomy, the MAG FEM_B0920 was identified by GTDB-Tk as *Dolichospermum* sp000312705. Furthermore, the *Dolichospermum* genome recovered from the Lake Garda bloom shared the highest average identities (ANI_b_ > 0.960) with the group of genomes included in GTDB R220 under *Dolichospermum* sp000312705, which corresponded, according to NBCI taxonomy, to several species mostly assigned to *Dolichospermum* spp. and *Anabaena* spp., as well as *D. lemmermannii, Dolichospermum flos-aquae* (Bornet and Flahault) P. Wacklin, L. Hoffmann, and Komárek and *Dolichospermum circinale* (Rabenhorst ex Bornet and Flahault) Wacklin, Hoffmann, and Komárek (Table 3 and Table S1). Specifically, *D. lemmermannii* CS-548 isolated from Lake Edlandsvatnet showed an ANI_b_ value of 0.966. The genome recovered from the Lake Superior bloom (*Dolichospermum* sp. SB001; not included in Table 3) showed an ANI_b_ value of 0.982.

**Table 3.** Average Nucleotide Identity (ANI) values between the *Dolichospermum lemmermannii* FEM_B0920 (GCA_037075685.1) and *Dolichospermum* genomes from GTDB, calculated using three different ANI formulations (see text).

ANI_b_	OrthoANIu	fastANI	GTDB taxonomy	NCBI taxonomy	Accession
0.979	0.979	0.978	*Dolichospermum* sp000312705	*Anabaena* sp. AL09	GCA_001672255.1
0.977	0.977	0.976	*Dolichospermum* sp000312705	*Anabaena* sp. LE011-02	GCA_001672225.1
0.969	0.968	0.966	*Dolichospermum* sp000312705	*D. circinale* CS-547	GCA_028329755.1
0.967	0.965	0.963	*Dolichospermum* sp000312705	*Dolichospermum* sp. UHCC 0260	GCA_009711985.1
0.967	0.964	0.966	*Dolichospermum* sp000312705	*Dolichospermum* sp. WA123	GCA_018447775.1
0.966	0.964	0.963	*Dolichospermum* sp000312705	*D. lemmermannii* CS-548	GCA_028330815.1
0.966	0.965	0.960	*Dolichospermum* sp000312705	*Dolichospermum* sp. UHCC 0299	GCA_009711965.1
0.966	0.963	0.962	*Dolichospermum* sp000312705	*Dolichospermum* sp. UHCC 0406	GCA_009712025.1
0.966	0.964	0.962	*Dolichospermum* sp000312705	*D. flos-aquae* CCAP 1403/13F	GCA_012516395.1
0.966	0.965	0.962	*Dolichospermum* sp000312705	*Dolichospermum* sp. DET73	GCA_017355625.1

Only results with ANI_b_ values ≥ 0.965 were included. Descending order of values according to ANI_b_. All the other *Dolichospermum* sp000312705 not included in this table have ANI_b_ values > 0.960.

### Phylogenomic analyses

**Figure 1.**
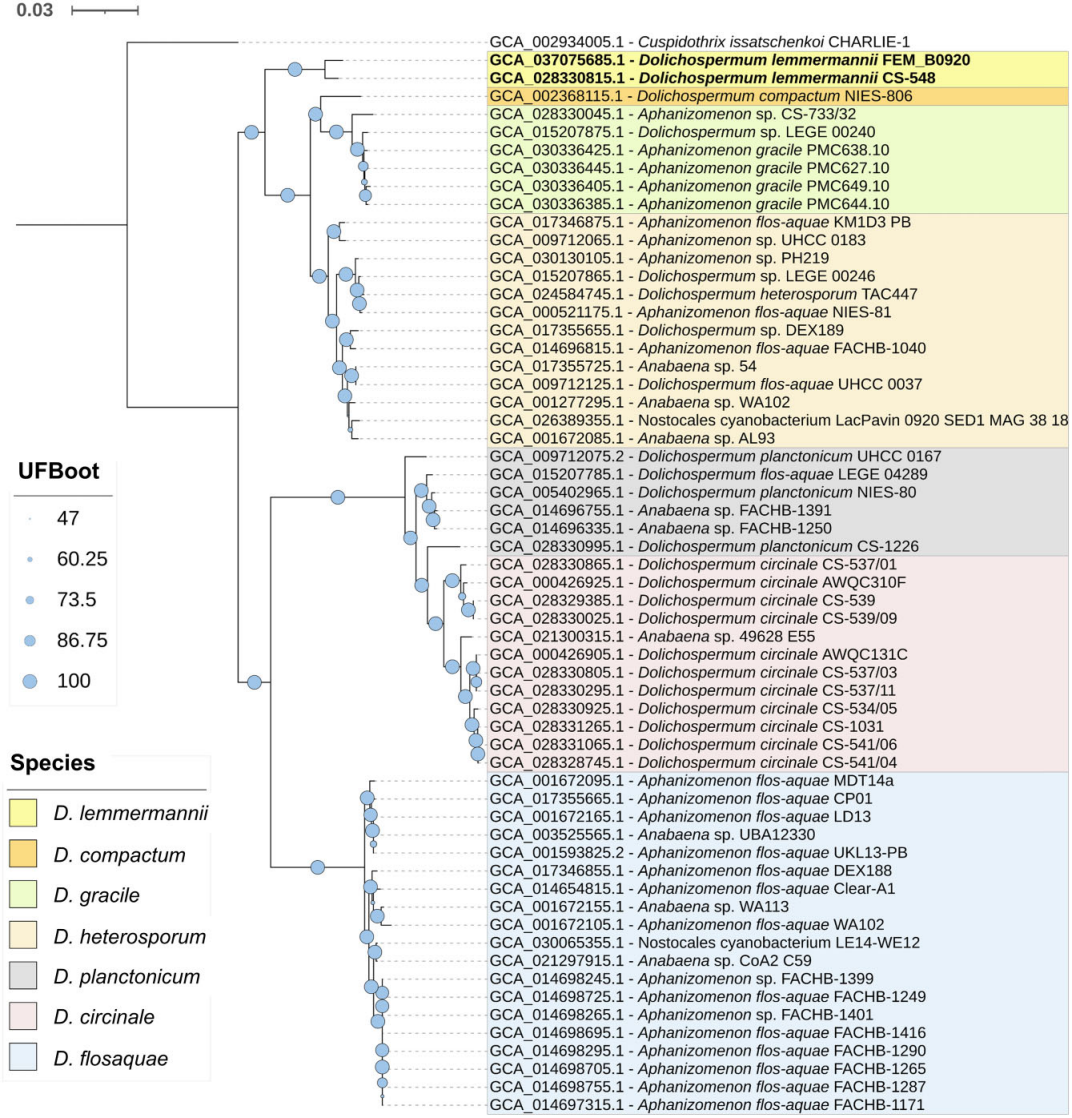
Phylogenomic tree of *Dolichospermum lemmermannii* FEM_B0920 together with several *Dolichospermum* species of the ADA group (*Anabaena, Dolichospermum* and *Aphanizomenon*) available in the Genome Taxonomy Database (GTDB). All genome names, strain identifiers and accession numbers are taken from the NCBI taxonomy. Species names are highlighted and grouped in different colors and correspond to the NCBI (*D. lemmermannii*; in bold) and GTDB taxonomy (*D. compactum, D. gracile, D. heterosporum, D. planctonicum, D. circinale*, and *D. flosaquae*) (see legend). The tree was rooted with *Cuspidothrix issatschenkoi* CHARLIE-1 as an outgroup. UFBoot, Ultrafast bootstrap values. The scale bar indicates the number of substitutions per site. Information on the individual assembled genomes is given in Table S1.

In the phylogenomic tree, all the genomes classified at the species level following the GTDB (*D. flosaquae, D. circinale, D. heterosporum, D. gracile*, and, partly, *D. planctonicum*) and NCBI taxonomy (*D. lemmermannii*) showed a clear separation into different clades (Fig. 1). *D. gracile* showed a relationship with the sole representative of *D. compactum*, but at a lower level of identity (ANI_b_ < 0.94) compared to intraspecific differences. Excluding *D. planctonicum*, all groups containing distinct species in compact clades were supported by UFBoot values > 95%.

**Figure 2.**
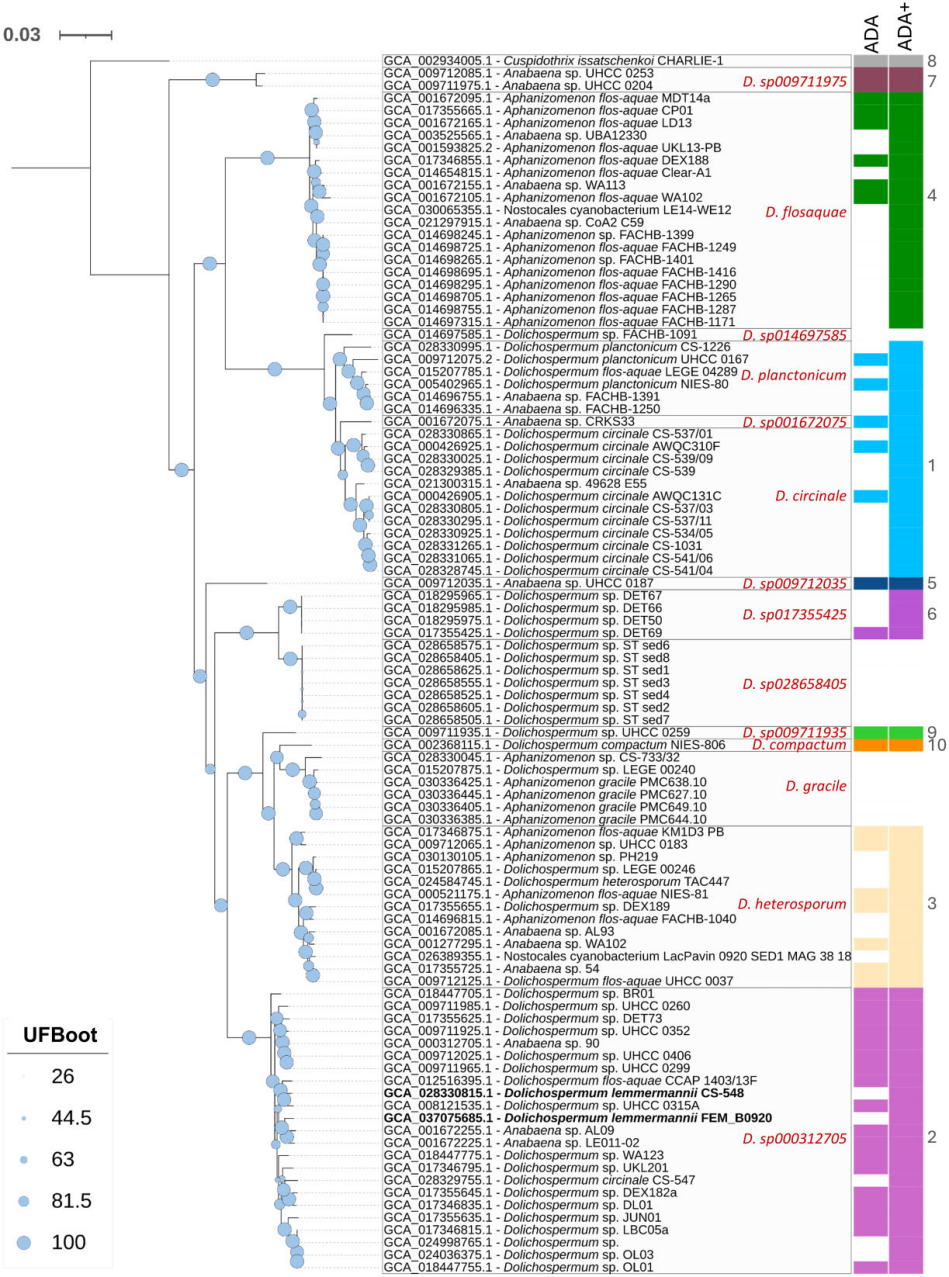
Phylogenomic tree of *Dolichospermum lemmermannii* FEM_B0920 and *Dolichospermum* taxa classified at either genus or species level available in the Genome Taxonomy Database (GTDB). All genome names, strain identifiers and accession numbers are from the NCBI taxonomy. For each clade, the names in red indicate the classification given by the GTDB taxonomy (excluding the *Dolichospermum* FEM_B0920 genome, not included in GTDB). ADA classifications are indicated by different colour codes; ADA and ADA+ refer to the classifications given in Driscoll et al. (2018) and Dreher et al. (2021), and estimated in this paper based on membership in the same clade, respectively. In ADA-2, the *Dolichospermum* genomes classified as *D. lemmermannii* in the NCBI taxonomy are highlighted in bold. The tree was rooted with *Cuspidothrix issatschenkoi* CHARLIE-1 as an outgroup. UFBoot, Ultrafast bootstrap values. The scale bar indicates the number of substitutions per site. Information on the individual assembled genomes is given in Table S1.

The phylogenomic analysis calculated using all the *Dolichospermum* genomes confirmed the results obtained with the analysis based on the species (Fig. 2). The close affinity of the two *D. lemmermannii* FEM_B0920 and CS-548 NCBI genomes to the *Dolichospermum* sp000312705 GTDB group (Table 3 and Table S1) was confirmed by the complete phylogenomic analysis, which showed the inclusion of these genomes in a unique compact cluster. While the ANI_b_ values between the Lake Garda genome (FEM_B0920) and all other genomes in this clade were always greater than 0.96, the ANI_b_ values calculated considering all other genomes not included in the *Dolichospermum* sp000312705/*D. lemmermannii* clade were always lower than 0.91.

The main clades, which included the major GTDB *Dolichospermum* taxa, were all assigned to different ADA representatives (Driscoll et al. 2018, Dreher et al. 2021) (“ADA” column in Fig. 2). This allowed the remaining untagged taxa to be assigned to distinct ADAs associated with the respective clades (“ADA+” column in Fig. 2 and Table S1), i.e. ADA-1 (mostly *D. circinale* and *D. planctonicum*; minimum ANI_b_ between species of the clade = 0.939), ADA-2 (*Dolichospermum* sp000312705/*D. lemmermannii*; minimum ANI_b_ = 0.959), ADA-3 (*D. heterosporum*; minimum ANI_b_ = 0.955), ADA-4 (*D. flosaquae*; minimum ANI_b_ = 0.978) and ADA-6 (*Dolichospermum* sp017355425; minimum ANI_b_ = 0.999). Within ADA-1, the ANI_b_ calculated separately for the species belonging to *D. planctonicum* and *D. circinale* showed higher minimum values, i.e. 0.947 and 0.946 (0.956 excluding *Anabaena* sp. CRKS33), respectively, than that calculated for the whole group of ADA-1 species. The remaining ADAs were less represented in the GTDB database.

### Relevant genes in the *Dolichospermum* FEM_B0920 MAG

PGAP identified 4439 protein-coding genes, 40 tRNAs, and complete sequences of 5S rRNA (3), 16S rRNA (1), and 23S rRNA (1) in the genome of *Dolichospermum* FEM_B0920 (Table 2). Excluding an uncultured bacterium and besides *D. lemmermannii* FEM_CADL9 (Table 4), the 16S rRNA gene was 100% identical, with query cover (QC) values 97%–99%, to several strains of *D. lemmermannii* isolated from Lake Garda and other deep lakes south of the Alps. In addition to *D. flos-aquae* CCAP 1403/13F (pident 99.26%; Table 4), the 23S rRNA gene showed percentage identity (pident) values > 98.5% (QC 100%) with six *Dolichospermum* and *Anabaena* taxa included in the *Dolichospermum* sp000312705 GTDB taxonomy (cf. Table 3 and Fig. 2). Comparable results (pident 98%–100% and QC 100%) were found for the three 5S rRNAs copies (111–112 bp). Of functional and taxonomic relevance, the top hit percentage identities of *rbcX* (assembly chaperone of ribulose-bisphosphate carboxylase/oxygenase, Rubisco) and *rpoB* (RNA polymerase B subunit) genes detected in the FEM_B0920 genome corresponded (QC 100%) to *Dolichospermum lemmermannii* NIVA-CYA 281/1 (99.5%) and *Dolichospermum* sp. LBC05a (*Dolichospermum* sp000312705) (99.3%), respectively. For *rpoB*, the sequence identified in the FEM_B0920 genome was 100% identical to some of the shortest sequences (575 bp) obtained by Sanger sequencing from strains of *D. lemmermannii* isolated from Lake Garda and other European lakes (Salmaso et al. 2015b, Capelli et al. 2017). These taxonomically relevant sequences are reported in Table S2.

**Table 4.** Megablast analysis of genes of taxonomic relevance and genes involved in geosmin biosynthesis from *Dolichospermum lemmermannii* FEM_B0920.

Gene	Q. Len. (bp)	QC (%)	Pident (%)	Acc. Len. (bp)	BLAST hit accession	NCBI taxonomy	Met.
16S rRNA	1489	99	100	1477	LT671839.1	*D. lemmermannii* FEM_CADL9	PGAP
23S rRNA	2832	100	99.26	genome	CP051206.1	*D. flos-aquae* CCAP 1403/13F^1^	PGAP
*rbcX*	430	100	99.53	808^2^	Z94883.1	*D. lemmermannii* NIVA-CYA 281/1	PGAP
*rpoB* ^3^	3339	100	99.25	genome	CP050882.1	*Dolichospermum* spp. LBC05a^4^	PGAP
Geosmin synthase	2274	99	98.23	genome	CP099464.1	*D. heterosporum* TAC447	AS

*rbcX*, RuBisCO chaperone RbcX encoding gene; *rpoB*, RNA polymerase B subunit gene; Q. Len., query length; QC, query coverage; Pident, % identity; Acc. Len., accession length; Met., genome mining method: PGAP, NCBI Prokaryotic Genome Annotation Pipeline; AS, antismash. Sequences are reported in Table S2.

1 Assembly GCF_012516395.1 (GTDB taxonomy, *Dolichospermum* sp000312705).

2 Subject sequence including *rbcL* and *rbcX* genes.

3 blastn, pident 100% and QC 17% to several strains of *D. lemmermannii* isolated and analysed (Sanger sequencing) from the large lakes south of the Alps (e.g. LN871475.1).

4 Assembly GCA_017346815.1 (GTDB taxonomy, *Dolichospermum* sp000312705).

Following the KEGG analysis, the complete and incomplete pathway modules found in the *D. lemmermannii* FEM_B0920 MAG were represented by the metabolism of cofactors and vitamins (42), amino acid metabolism (38), carbohydrate metabolism (32), and energy metabolism (27), whereas the remaining pathways included from 1 to 11 modules ( Table S3A). A number of modules contained reactions essential for the central metabolism of photosynthetic cyanobacteria, including oxygenic photosynthesis (photosystems II and I; modules M00161 and M00163), beta-carotene biosynthesis (M00097), and phycobilisomes (allophycocyanin and phycocyanin/phycoerythrocyanin, but not phycoerythrin; Fig. S4), the reductive pentose phosphate cycle (Calvin cycle) (M00165), the TCA (tricarboxylic acid - Krebs) cycle (M00009) and glycolysis (M00001) (Table S3A).

Specific pathways were relevant for diazotrophic blooming species. Besides assimilatory nitrate reduction (M00531, which included the *narB* nitrate reductase and *nirA* nitrite reductase genes), nitrogen metabolism was sustained by nitrogen fixation (M00175) (Fig. S5). Specifically, the annotation by Bakta revealed the presence of different *nif* genes involved in the fixation of atmospheric nitrogen (i.e. *nifB, D, E, H, J, K, N, S, T, U, V, W, and X*). No modules associated with dissimilatory nitrate reduction, denitrification, nitrification, and anammox were identified (Fig. S5).

Several protein components of ATP-binding cassette (ABC) membrane transporters for a wide range of nutrients, micro-elements, and organic molecules were identified in the *D. lemmermannii* FEM_B0920 genome (Fig. S6). To support the intracellular assimilatory N-reduction and N-uptake, genes encoding a nitrate/nitrite transporter were present (*nrtABC*), complemented by ammonium uptake (K03320; *amt* gene). A bicarbonate transporter (CmpABCD) was part of the carbon-concentrating mechanism (CCM). Other set of genes encoded proteins for the selective transport of molybdate and organic molecules, such as the polyamines spermidine/putrescine, osmoprotectants, oligosaccharides (several with incomplete paths), polyols and lipids, and lipopolysaccharides. Supporting the assimilatory sulfate reduction (M00176), besides ABC transporters for sulfate/thiosulfate and alkanesulfonate (as an additional source of S), a gene encoding non-ABC lower affinity sulfate transport was detected (K03321, sulfate permease). FEM_B0920 included genes encoding active transporters for phosphate and organophosphorus compounds (phosphonate), as well as amino acids and the rich-N urea, CO(NH_2_)_2_. The Pst system (phosphate ABC transporter; Fig. S6) was complemented by Pho regulon components (PhoHURB; K06217, K02039, K07636, and K07657) involved in the regulation of P-uptake. A group of genes was involved in the synthesis of ABC transporters targeting growth elements such as, besides molybdenum (in the form of molybdate), zinc, cobalt, and nickel. Though potentially biosynthesized by FEM_B0920 (M00950), specific proteins were potentially encoded for the transport of biotin (vitamin B_7_).

The presence of genes encoding ferrous iron transport proteins A (FeoA, K04758) and B (FeoB, K04759) were also identified.

A few enzymes (K04564, K07217, K24157, K24158, K00799, K01920, K00383, and K09825) were identified that encode superoxide dismutase, catalase, catalase-peroxidase, peroxiredoxins and other enzymes involved in the regulation of oxidative stress response mechanisms required for the removal of reactive oxygen species (ROS), i.e. superoxide (O_2_^−^) and hydrogen peroxide (H_2_O_2_) produced as byproducts by photosynthesis.

The essential role of cofactors and vitamins in various biochemical reactions essential for the maintenance of cellular functionality was expressed by the presence of several complete or nearly complete modules associated with their biosynthesis; among others, and in addition to biotin/vitamin B7, vitamins B1 (thiamine), B2 (riboflavin), B5 (Pantothenate), B6 (Pyridoxal-P), and B12 (Cobalamin).

In the KEGG database, specific modules describing the gene cluster involved in the gas vesicle biosynthesis are not present. Different *gvp* genes in the *D. lemmermannii* FEM_B0920 genome were however identified by specific K-numbers (K23262) and by Bakta annotation.

KEGG annotation of the *D. lemmermannii* CS_548 genome produced results that were almost indistinguishable from those obtained with the *D. lemmermannii* FEM_B0920 annotation (Tables S3A–B and Fig. S6).

### Genes potentially involved in the synthesis of bioactive peptides

The antismash analysis allowed the detection of distinct secondary metabolite regions (Table 5). These included regions involved in the physiology of heterocytous N-fixing cyanobacteria (heterocyst glycolipids) and in the biosynthesis of GEO (Table S2). No regions involved in the biosynthesis of the “conventional cyanotoxins” commonly identified in Lake Garda, such as MCs and ATXs (Cerasino and Salmaso 2012), were detected. On the contrary, new bioactive secondary metabolites, some with potential inhibitory/toxic activity, belonging to the classes of non-ribosomal peptides (NRP) and ribosomally produced natural products (RiPP) were identified (Table 5). The first class included anabaenopetins, scytocyclamides (laxaphycins) and a mycosporine-like compound; varlaxin was detected with very low similarity. The second class included the anacyclamides.

**Table 5.** Major secondary metabolites identified by antismash on *Dolichospermum lemmermannii* FEM_B0920.

BGC type	Region length (bp)	Most similar known cluster	Similarity	Property
NRPS	8935	anabaenopeptin/nostamide—NRP	44%	Phosphatases and proteases inhibition/antimicrobial, cytotoxic
NRPS	4026	anabaenopeptin—NRP	42%	Phosphatases and proteases inhibition
NRPS	13 791	scytocyclamides—NRP+Polyketide	38%	Antifungal activity, cytotoxicity
NRPS	37 118	varlaxin—NRP	9%	Aeruginosin-type inhibitors of human trypsins
Cyanobactin	12 623	anacyclamide—RiPP:Cyanobactin	35%	Antibacterial
Cyanobactin	4478	anacyclamide—RiPP:Cyanobactin	28%	Antibacterial
hglE-KS, T1PKS	32 443	heterocyst glycolypids	85%	Involved in heterocyte glycolipids biosynthesis
hglE-KS	37 883	heterocyst glycolypids	57%	Involved in heterocyte glycolipids biosynthesis
Terpene	22 274	geosmin—Terpene	100%	Impart earthy/musty taste and odor
Mycosporine-like	16 006	hexose-palythine-serine/hexose-shinorine—NRP	28%	MAAs protection against UV damage

BGC, biosynthetic gene cluster; NRPS, non-ribosomal peptide synthase; PKS, polyketide synthase; hglE-KS, heterocyst glycolipid synthase-like PKS; RiPP, ribosomally synthesized and post-translationally modified peptide product. Only secondary metabolite regions showing similarity to a known biosynthetic cluster are shown. Similarity indicates the percentage of genes within the closest known compound that has a significant BLAST hit to genes within the current region (Blin et al. 2023).

Besides antismash, the absence of gene clusters encoding microcystins and anatoxins in the FEM_B0920 genome was confirmed by the negative results obtained with ISeqDb searching for the presence of *anaC, anaF, mcyB, sxtA*, and *cyrJ*. The genes *mcyD* and *mcyE* were detected with two short sequences (197 and 128 bp, pident 95.9% and 99.2%) similar to fragments characterized by MITE (miniature inverted–repeat transposable elements) insertion (Fewer et al. 2011). These short fragments were also detected in other *Dolichospermum* genomes considered in this work.

Compared to the *D. lemmermannii* FEM_B0920, the genome of *D. lemmermannii* CS-548 isolated from Lake Edlandsvatnet, Norway, showed the presence of microcystin genes. Furthermore, besides MC, the ability of this genus to potentially synthesize ATX, STX, and CYN was well documented after the analyses by antismash (Table S1). Some apparent patterns were distinguishable, i.e. a broad exclusive presence of genes encoding MC in ADA-2; a broad exclusive presence of genes encoding ATX in ADA-3 (and ADA-8, the outgroup); the presence of genes encoding STX in *D. gracile* and in one genome in ADA-1; excluding two annotations with weak support, exclusive presence of genes encoding CYN in ADA-6. Genes encoding anabaenopeptins (APs) were present in ADA-2 and ADA-3, *D. gracile*, and all the *Dolichospermum* sp028658405 genomes. Genes encoding GEO were well represented in ADA-1 and ADA-3, and only sporadically in ADA-2. Noteworthy is the absence of all analysed genes encoding MC, ATX, STX, CYN, APs, and GEO in the genome of. *D. flosaquae* (ADA-4) and, excluding GEO, *D. planctonicum* (ADA-1). In ADA-1, in addition to the detection of a genome containing STX genes found by antismash, the analysis of two *D. circinale* strains was positive for the biosynthesis of STX using analytical methods (Beers 2020), while five strains were positive for the presence of the gene encoding sxtA (Table S1).

### Metagenomic analyses of the bacterial community

The MetaPhlan analysis classified the 30% of the quality-filtered reads as representative of the surface sample (scum). Cyanobacteria (28.4%) were mainly represented by *Dolichospermum* (28.1%), over the remaining bacterial classes, mainly represented by Gammaproteobacteria and Alphaproteobacteria. Besides *Dolichospermum*, the other cyanobacteria were detected with relative abundances well below 0.3% and were represented by *Microcystis aeruginosa, Tychonema bourrellyi* (reported as *Microcoleus bourrellyi* in the GTDB taxonomy), and picocyanobacteria (*Synechococcus lacustris* and *Cyanobium usitatum*; Cabello-Yeves et al. 2018). Exceedingly rare reads included *Dolichospermum* spp., *Aphanizomenon* spp., *Planktothrix* spp., and *Cuspidothrix issatschenkoi*.

MAGs recovered from the binning of the contigs included representatives of the classes found by MetaPhlan, mostly belonging to Gammaproteobacteria and Alphaproteobacteria (Table S4). Representatives of the first group included *Acidovorax* and *Rubrivivax*, while the second group included *Tagaea, Rhabdaerophilum* and *Sphingorhabdus*. No genes involved in the biosynthesis of cyanotoxins and GEO were found in any of the identified bacterial contigs.

The whole set of raw contigs, including those unbinned and not included in any MAGs and those with a length < 1000 bp excluded from the binning procedure were analysed for the presence of MC, ATX, STX, and CYN, as well as GEO-encoding genes. For anatoxin-a, *anaC* and *anaF* were identified with sequences of 113 and 346 bp, respectively, showing 100% similarity to the corresponding genes in the anatoxin-a-producing *Tychonema bourrellyi* B0820 isolated from Lake Garda (Salmaso et al. 2023). In addition to the sequences including the MITE insertion (previous section), further fragments of *mcyB* (~200 bp) and *mcyE* (around 370 bp) were identified in other contigs not included in the MAGs with pident 95%–100% to uncultured cyanobacteria and *Microcystis*. Conversely, no other fragments of the *sxtA, cyrJ*, and *geoA* genes were identified in the entire contig set, except for *geoA* identified in *Dolichospermum* FEM_B0920.

### AMR genes

Functional annotation of *D. lemmermannii* FEM_B0920 by Bakta and/or PGAP identified a tetracycline resistance protein, class C, and the multidrug resistance protein MexB. After protein BLAST, the sequences showed 100% QC and up to 100% and 99.6% similarity to the MFS transporter (Pasqua et al. 2019) of several *Anabaena/Dolichospermum* species and efflux RND transporter permease subunit (Nappier et al. 2020, Hwengwere et al. 2022, Aguiló-Nicolau et al. 2023), respectively. KEGG annotation found one ortholog (K17836) associated with the beta-Lactam resistance (Bush 2013). No AMR genes were identified by ABRicate in the *D. lemmermannii* FEM_B0920 and in the complete set of raw contigs, using the adopted thresholds for minimum identities and coverage.

## Discussion

The metagenomic analysis of a surface sample collected during a cyanobacterial bloom identified in Lake Garda in late summer 2020 allowed to confirm the nature of the organism responsible for the episode and to functionally characterize the population. The analyses allowed to clarify the phylogenomic position of *D. lemmermannii* in relation to other species of the same genus and ADA group and to interpret the adaptive ecological traits in relation to the range of primary and secondary metabolites potentially produced by the population involved in the bloom.

### Environmental conditions during the bloom

*D. lemmermannii* blooms in the lake district south of the Alps were first recorded in Lake Garda at the turn of the 1980s and 1990s. Gradually, blooms also appeared in the other large and deep lakes south of the Alps, namely Lakes Iseo, Como, Maggiore, and Lugano (Callieri et al. 2014, Funari et al. 2014). In Lake Garda, the whole set of microscopic and genetic analyses carried out since the 1990s confirmed the presence of a unique Nostocales in the cyanobacterial communities involved in the blooms (Salmaso et al. 2015b, Capelli et al. 2017).

The long-term historical colonization of *D. lemmermannii* in Lake Garda was investigated by direct counting of subfossil akinetes identified from sediment cores and by estimating the nature and abundance of filaments germinated from subfossil viable akinetes by light microscope and genetic analyses (Salmaso et al. 2015a). The application of this complementary approach allowed to identify the onset of colonization around the mid-1960s, when the lake showed a shift from ultra-oligotrophy / oligotrophy to oligo-mesotrophy (Milan et al. 2015).

The analysis of long-term limnological data collected in Lake Garda since the 1990s showed that *D. lemmermannii* filaments always developed during the warmest months, with temperatures >15°C and abundances generally <40 mm^3^ m^−3^ in the layer 0−20 m. Bloom formation during summer and early autumn was favoured by high temperatures, high water stability and calm weather (Salmaso et al. 2015b). Given the extremely low biomass of *Dolichospermum* in the epilimnetic layer, blooms were caused by the rapid upward movement and accumulation of filaments towards the surface, rather than by in situ growth. The development of this species during the warmest months coincided with the periods of minimum availability of dissolved nitrogen concentrations, which were generally <100–150 µg N L^−1^. These conditions were the same as those recorded during the bloom observed in September 2020. In particular, the low concentrations of SRP and TP (<5 and < 10 µg P L^−1^, respectively) precluded the development of high phytoplankton biomasses in the first 20 m, whereas the low DIN concentrations (below 50 µg N L^−1^) recorded in the first 10 m would indicate a state of nitrogen limitation, potentially favouring heterocytous nitrogen-fixing cyanobacteria (Schindler et al. 2016, Maberly et al. 2020, Chorus and Spijkerman 2021). Due to the low biomass associated with surface blooms and the strong constraints imposed by low nutrient concentrations on cyanobacterial development in the epilimnion, these episodes have been termed “oligotrophic blooms” (Salmaso et al. 2015b and references therein).

### Taxonomic position within the *Dolichospermum* species group

Genomic analyses were performed using *Dolichospermum* genera and species classified by the GTDB initiative, which uses a standardized microbial taxonomy based on genome phylogeny, with genomes obtained from NCBI RefSeq (Reference Sequence Database) and GenBank (Parks et al. 2022). The GTDB taxonomy is based on genome trees inferred from aligned concatenated sets of single-copy marker proteins for Bacteria and Archaea and ANI comparisons, while the LPSN (List of Prokaryotic names with Standing in Nomenclature) (Parte et al. 2020) is used for nomenclatural reference and to establish naming priorities and nomenclature types. In this respect, the phylogenomic and ANI comparative approaches used to define ADA groups (species) are similar to those used by the GTDB, and the two classifications provide comparable results in defining clades. The use of genomic-based approaches is the only objective way to disentangle a legacy of names adopted by different laboratories to classify Nostocales. Consistent with the GTDB approach (Parks et al. 2020), there is a convergence of opinion on the possibility of homogenizing and updating the species names of Nostocales included in the same clades and ADA groups (Österholm et al. 2020, Dreher et al. 2021). In this direction, the GTDB taxonomy represents an important conceptual and practical step, but it is open to updates, as species representatives are re-evaluated with each GTDB release. At present, the main limitations are due to the poor representation in the taxonomic databases (International Nucleotide Sequence Database Collaboration, and GTDB) of several well-documented genomes of species of Nostocales (and cyanobacteria in general), which still represents an obstacle to the correct determination of species of difficult attribution (e.g. Woodhouse et al. 2024) and to the completion of the ADA taxonomy based on the adoption of genomic criteria. In addition, most genomes were obtained from only a few countries, which may have introduced a geographical bias into the results of the taxonomic and annotation analyses. For example, although well characterized, the ADA-4 clade, which included several species of *Aphanizomenon flos-aquae* Ralfs ex Bornet and Flahault, was reclassified under the name *Dolichospermum flosaquae* in the GTDB taxonomy. At the same time, the available *Dolichospermum flos-aquae* genomes in the NCBI database were included, according to the genomic criteria, in three different clades (i.e. *Dolichospermum* sp000312705/ADA-2, *D. heterosporum*/ADA-3 and *D. planctonicum*/ADA-1). These two species are validly published according to the International Code of Botanical Nomenclature , have different morphologies (Komárek 2013) and are capable of producing a different range of toxins (Bernard et al. 2017). Furthermore, the ADA7 at the extreme end of the tree (Fig. 2) is composed of two benthic strains originating from the brackish waters of the Baltic Sea, questioning their inclusion in the genus *Dolichospermum* (see Österholm et al. 2020). Clarification of the taxonomic position of *Dolichospermum* within this classification scheme requires better coverage of the constituent genomes. Similar considerations apply to the other groups in the tree, including the ADA-1 clade, which, as already suggested by Driscoll et al. (2018) and Dreher et al. (2021), could be split into two distinct species/subspecies, consistent with the discrimination of the two sets of genomes originally classified under *D. planctonicum* and *D. circinale* (Komárek 2013).

The two *D. lemmermannii* genome assemblies classified in the NCBI taxonomy (FEM_B0920 and CS-548) showed high genomic similarity (ANI_b_ > 0.96) with a large group of *Dolichospermum* and *Anabaena* species, which are collectively grouped within the *Dolichospermum* sp000312705 taxon defined in the GTDB taxonomy and within the ADA-2 group. Overall, the results would suggest a relationship between the taxa represented in this group and *D. lemmermannii*.

### Functional annotation

The two photosystems and their associated reactions, the reductive pentose phosphate cycle, the TCA cycle and glycolysis may be considered the major core pathways that characterize cyanobacterial metabolism. Other more specific metabolic pathways are differentially present in cyanobacteria and closely associated with selective traits that promote cyanobacterial growth and bloom formation (Cao et al. 2020). In this regard, in the FEM_B0920 MAG, specific traits were associated with phenological and physiological characteristics of bloom-forming Nostocales.

Related to photosynthetic processes, the presence of genes encoding phycocyanin and allophycocyanin, which absorb far-red and red-orange light, is consistent with the development of the *D. lemmermannii* population in the surface epilimnetic waters of Lake Garda (Salmaso et al. 2015b). Phycoerythrin is mostly found in species that use the green-yellow region of the spectrum in low-light deeper waters and in species forming metalimnetic layers (Knapp et al. 2021).

Carbon, hydrogen, nitrogen, oxygen, phosphorus and sulfur are the six bulk macronutrients (CHNOPS) sustaining life (Fagerbakke et al. 1996, Remick and Helmann 2023). Among the CHNOPS elements, N and P are often present at low environmental concentrations and require targeted cellular transporters for their uptake (Reynolds 2006, Yang et al. 2022). Similarly, under high photosynthetic activity and high pH conditions, CO_2_ and HCO_3_^−^ decrease in favour of CO_3_^2−^, which is not directly utilized by microalgae, leading to C-limited conditions (Stumm and Morgan 1996, Wetzel 2001).

The presence of nitrogen fixation genes in the FEM_B0920 genome suggested the potential ability of the *D. lemmermannii* population to fix atmospheric nitrogen. Although the current practice for computational prediction of N fixation is based on the presence of the *nifH* and/or *nifD* genes (Dos Santos et al. 2012), it was suggested that the presence of a minimum set of six genes encoding structural and biosynthetic components, i.e. NifHDK and NifENB, should be verified, as in the FEM_B0920 MAG. At the ultrastructural level, the potential for N-fixation was confirmed by the identification of the complex of genes encoding heterocyte glycolipids (Garg and Maldener 2021, Pérez Gallego et al. 2023). The presence of heterocytes in the filaments of *Dolichospermum* observed in Lake Garda is quite common, see, e.g. Fig. S3 in Salmaso et al. (2015b), but their quantitative estimation was never performed in the sample collected in this or previous blooms. Given the evolutionary establishment and success of nitrogen fixation in bacteria, the physiological and competitive benefits are likely to outweigh the energetic costs. Nevertheless, while experimental measurements have assessed quantifiable rates of N-fixation in several lakes at different levels of environmental nitrogen (Natwora and Sheik 2021, Marcarelli et al. 2022, Ehrenfels et al. 2023) and nitrogen and CO_2_ concentrations (Kramer et al. 2024), no experimental evidence has been collected by performing nitrogen fixation assays in Lake Garda. On the other hand, in addition to exogenous inorganic (nitrate, nitrite and ammonium) transporters, the ability for organic nitrogen uptake was identified in the FEM_B0920 MAG, suggesting the potential scavenging of additional sources of N compounds during the nutrient-poor summer period. Various types of amino acids, urea, putrescine and spermidine are common organic nutrient sources produced by the planktic community that can be used by microorganisms as a source of carbon and nitrogen. The elevated dissolved organic nitrogen levels observed in Lake Superior during the blooms of *D. lemmermannii*, coupled with a decrease in nitrate, indicated that nitrogen species conversion and cycling may have played a significant role in maintaining the blooming population (Sterner et al. 2020, Sheik et al. 2022). In Lake Garda, due to the typically low epilimnetic microalgal biomass observed during the summer months, the contribution of the external organic nutrient sources remains to be quantified.

The presence of genes for the potential active uptake of P and bicarbonates is similarly indicative of adaptations to low-nutrient conditions during the summer months and blooms. In bacteria, the synthesis of the Pst phosphate transport system is promoted under low P-concentrations, as demonstrated in *Nostoc punctiforme* Hariot under P-starvation conditions (Hudek et al. 2016). The Pho regulon is responsible for sensing environmental phosphate levels and is therefore critical in regulating adaptive responses to P limitation, particularly given its activity under low-P conditions (Santos-Beneit 2015, Zhang et al. 2024). Additional sources of P could potentially be provided by the uptake of organophosphorus compounds, e.g. phosphonates (Xiao et al. 2022), although known genes involved in subsequent mineralization after uptake (such as CP lyase; *phnJ*, K06163) were not identified in the FEM_B0920 MAG. As only a few cyanobacterial species possess genes encoding C-P lyase, the mineralization of phosphonate by the phycosphere community was described as an additional mechanism enabling organic phosphorus scavenging (Zhao et al. 2023).

Five different inorganic carbon uptake systems have been identified in different model cyanobacteria (Hagemann et al. 2021). The *cmpABCD* cluster in the FEM_B0920 MAG encodes an ATP-binding cassette transporter involved in HCO_3_^−^ uptake (Maeda et al. 2000, Koropatkin et al. 2007). This operon is part of the CCMs in cyanobacteria, potentially mitigating the decrease in CO_2_ when pH is progressively higher than 8. Inorganic carbon transporters allow high levels of HCO_3_^−^ to accumulate inside cells, especially when free CO_2_ is very low, and the cells are mainly consuming bicarbonate from the medium. When accumulated into the cell, bicarbonate penetrates into carboxysomes, where it is dehydrated to CO_2_ in proximity to RubisCO (Burnap et al. 2015).

Sulfur is an essential component of the amino acids cysteine and methionine and an essential constituent of several cellular cofactors (Scott et al. 2007). Sulfur limitation reduces cyanobacterial growth, alters the cellular ultrastructure and exerts inhibitory effects on photosynthesis (Kharwar et al. 2021). In addition to sulfate, the uptake of organosulfur compounds like alkanesulfonates is an additional or alternative sulfur source. Once inside the cell, the sulfonate group is converted to inorganic sulfate or sulfite by specific enzymes such as alkanesulfonate monooxygenase (*ssuD*; K04091) in the FEM_B0920 genome. Induction of high-affinity sulfate transporters is only activated under sulfate deficiency (Kharwar et al. 2021, Kharwar and Mishra 2024). According to Reynolds (2006), unlike C, N and P, sulphur is usually in excess relative to phytoplankton requirements, and sulfate normally saturates the S-uptake of algae down to concentrations of 4.8 mg SO_4_^2−^. In *Aphanothece* (*Anacystis*) *nidulans* P. Richter, Utkilen et al. (1976) and Green and Grossman (1988) reported half-saturation constants for sulfate uptake of 0.75 and 1.35 µM, indicating that, for this species, the transport of SO_4_^2−^ could be limited at low concentrations down to ca. 0.1 mg L^−1^.

Along with the presence of several transporters targeting growth microelements, the presence of several complete or nearly complete modules associated with the biosynthesis of cofactors and vitamins represented a crucial factor in ensuring a wide range of metabolic processes in a wide range of changing environmental conditions (Romine et al. 2017, Żymańczyk-Duda et al. 2022, Shah et al. 2024).

The surface bloom of *D. lemmermannii* was controlled by the biosynthesis of gas vesicles, which is mediated by several *gvp* genes (Walsby 1994, D'Alelio et al. 2011, Hill and Salmond 2020), some of which have been identified in the FEM_B0920 genome. Under calm conditions and with a high rate of gas vesicle formation, *D. lemmermannii* filaments can reach upward vertical velocities of up to 0.7–0.9 m h^−1^ (Walsby et al. 1991), thus explaining the sudden formation of scums. Under these conditions, with high solar radiation and O_2_ availability, high production of ROS can severely damage the functionality of cells (He and Häder 2002), making the removal of ROS via enzymatic reaction a key mitigating selective factor.

### AMR

The absence of ARGs in the *Dolichospermum* bloom may be related to the oligotrophic status of the lake. In the same lake district, Di Cesare et al. (2024) reported extremely low concentrations of antibiotics and other pharmaceuticals in the oligotrophic Lake Maggiore. The presence of ARGs in the *Dolichospermum* genome, as indicated by Bakta or KEGG, would require further analysis, considering a larger number of samples to be evaluated. This is particularly relevant as a study of 862 high-quality cyanobacterial genomes revealed a high diversity of ARGs, especially in Nostocales, which had the highest number of species with ARGs (67 out of 301) (Timms et al. 2023).

### Conventional and emerging secondary metabolites

The absence of gene clusters or single genes encoding MCs and ATX in the *D. lemmermannii* population that caused the Lake Garda bloom in 2020 fully confirmed previous studies carried out on several strains isolated from Lake Garda and other large lakes south of the Alps (Salmaso et al. 2015b, Capelli et al. 2017, Cerasino et al. 2017). The FEM_B0920 genome contained short fragments of *mcyD* and *mcyE* with a MITE insertion (Fewer et al. 2011); their presence could indicate inactivation of the *mcy* gene cluster by genetic rearrangement, but proper analysis of this topic would require dedicated and complete analyses of a representative number of genomes.

The low concentrations of ATX detected in the bloom of *D. lemmermannii* in Lake Garda were presumably produced by *T. bourrellyi*, which until now was the only ATX producer isolated in Lake Garda (Shams et al. 2015, Cerasino and Salmaso 2020, Salmaso et al. 2023). This was confirmed by the identification of *anaC* and *anaF* sequences in the complete set of contigs, with 100% similarity to *Tychonema bourrellyi*.

Several gene regions potentially involved in the biosynthesis of secondary metabolites have been identified in the *D. lemmermannii* FEM_B0920 genome. GEO is a well-known terpene volatile compound produced by a wide range of bacteria and cyanobacteria in terrestrial and aquatic environments giving soil and water an earthy odour. Although not toxic to humans via drinking water at environmentally relevant concentrations, GEO can lead to a loss of consumer confidence in water quality (Akcaalan et al. 2022, Manganelli et al. 2023). In this work, GEO encoding genes were detected in ADA-1 and ADA-3, and partly in ADA-2.

Some NRPs can be classified as emerging chemical contaminants, i.e. compounds that are not generally monitored and not subject to regulation, but which have the potential to have adverse effects on human health and ecosystems (Parida et al. 2021, Morin-Crini et al. 2022). Among these, anabaenopetins are a family of cyclic hexapeptides that have been identified in a large number of cyanobacteria (Sterner et al. 2020, Monteiro et al. 2021, Dreher et al. 2023, Zastepa et al. 2023). Congeners of APs have been shown to have inhibitory activity against phosphatases and proteases, but their potential effects on human health remain to be evaluated (Gkelis et al. 2015, Monteiro et al. 2021). Among NRPs, scytocyclamides are laxaphycins discovered in *Scytonema hofmannii* (Heinilä et al. 2020). Scytocyclamides and laxaphycins have shown significant antifungal activity, usually coupled with cytotoxic activity (Fewer et al. 2021), as well as toxicity against the crustacean *Thamnocephalus platyurus* (Darcel et al. 2021). Varlaxin is a new NRPS aeruginosin-type inhibitor of human trypsins (Heinilä et al. 2022). A congener of this metabolite showed inhibition of human prometastatic trypsin-3, making varlaxin a potential lead molecule for drug development (Heinilä et al. 2022). This BGC showed a broad presence in the *Dolichospermum* genomes (data not shown), although it was detected in strain FEM_B0920 with a very low similarity value.

Among RiPPs, cyanobactins may be involved in the competition between strains or act as antimicrobial agents against bacteria (Nowruzi and Porzani 2021). Mycosporine-like amino acids (MAAs) are produced by a variety of organisms to protect against ultraviolet (UV) damage (Chen et al. 2021). Although still controversial (Hu et al. 2015), the presence of MAAs was related to the protection against UV radiation during high solar irradiances (D'Agostino et al. 2016, Yang et al. 2018, Geraldes et al. 2020, Jacinavicius et al. 2021), such as those experienced during blooms (Zhang et al. 2022).

### Cyanotoxins and other encoding genes in *Dolichospermum* species

The distribution of genes encoding cyanotoxins, APs and GEOs showed a well-distinguishable pattern in each ADA clade, suggesting a substantial relationship between genome identities within an individual species and the biosynthesis of these secondary metabolites. This is in agreement with the results of Österholm et al. (2020). Genes or gene clusters encoding STX and CYN in *Dolichospermum* were investigated by Ledreux et al. (2010), D'Agostino et al. (2020), and Halary et al. (2023), and by Dreher et al. (2022), respectively, while genes encoding ATX were also investigated by Wood et al. (2007) and Rantala-Ylinen et al. (2011). Studies on MC-producing strains included, among others, Rouhiainen et al. (2004) and Dreher et al. (2019).

The ability to potentially synthesize specific cyanotoxins in specific phylogenomic clades has important implications for the expected impacts and potential risks associated with the development of ADA species. Nevertheless, the very limited geographical areas of origin of the genomes (in particular ADA-1/*D. circinale*, ADA-4 and ADA-6) and/or the under-representation of genomes in some ADA groups could introduce a bias in the representativeness of the results.

Obtaining reliable information on the potential of microorganisms to synthesize active biomolecules using genome mining techniques requires analyses to be performed on genomes that are as complete and uncontaminated as possible. When applied to fragmented or poor-quality genome assemblies, genome annotation tools can produce inconstant results (Skinnider et al. 2020). In this respect, while genome mining may provide a remarkable screening tool and an essential guide to assess the potential of specific cyanobacterial populations to synthesize a range of harmful compounds, a complete risk assessment procedure should always consider the inclusion of chemical analytical techniques.

### From genes to functions: extending the characterization of functional traits and competitive adaptations

The analysis of the genetic characteristics of cyanobacteria allows accessing explicit information on general metabolic pathways and specific adaptive and competitive physiological capabilities proper of particular groups or species/strains morphologically similar or undistinguishable but with different genetic and functional characteristics. From an ecological point of view, this represents a considerable step able to integrate and substantially widen the functional characterization of cyanobacteria and phytoplankton based on structural morphometric and morphological traits like, among others, cell size and shape, arrangement of cells, presence of mucilage and gas vesicles (B-Béres et al. 2024). Besides common functional traits represented by the central metabolism of photosynthetic cyanobacteria, a few distinctive and adaptive traits contributed to defining the factors promoting algal blooms in oligotrophic environments, including the presence of several high- and low-affinity transporters for macro-, micronutrients, and organic compounds; the possession of a gene pool for nitrogen fixation; the ability to control vertical position; adaptations to remove reactive oxygen species produced during photosynthesis; the ability to produce MAAs involved in UV protection of cells exposed to high irradiances. All these traits delineate the set of competitive functions that *D. lemmermannii* can potentially express in oligotrophic lakes.

## Conclusions

Cyanobacterial blooms pose a potential risk to human and environmental health and function. A reliable assessment of the risks associated with massive population development or physical accumulation of potentially toxigenic cyanobacteria requires a comprehensive assessment of the gene pool responsible for cyanotoxin production and metabolomic profiling. However, targeted analysis of individual cyanotoxins requires specific, separate laboratory protocols for both polymerase chain reaction and later Sanger sequencing, as well as individual metabolite characterization. In addition to being time-consuming and costly, this approach is generally directed towards the analysis of conventional cyanotoxins, without taking into account the high metabolomic diversity of cyanobacteria and thus ignoring other bioactive molecules and potential sources of risk. In this context, the determination of the draft genomes of the cyanobacterial and bacterial consortium provides rapid indications of both the taxonomic nature of the populations living in aquatic ecosystems and their functional profile, with a comprehensive analysis requiring a unique HTS run combined with bioinformatic analyses. The application of this approach to a *D. lemmermannii* bloom in Lake Garda allowed to evaluate the taxonomic position of this species within the GTDB and ADA classification schemes, identifying a clear cluster including *D. lemmermannii* within ADA-2, but with still many uncertainties in the definition of the whole ADA classification system due to many gaps in the coverage of species genomes in NCBI and GTDB. Genome mining allowed the discovery of a number of genes encoding specialized functions relevant to bloom-forming heterocytous Nostocales and a set of secondary metabolites previously unknown in populations of this species developing in the southern Alpine lake district. In addition to their taxonomic and ecological relevance, the results have management implications by challenging the completeness of analyses obtained using conventional approaches. In this context and considering that the functional analyses of genomes provide information on the presence and potential expression of genes, the results obtained should also be considered as an essential guideline to better address analytical efforts in the chemical analytical determination of metabolites of interest for potential effects on human health and the characterization of compounds of pharmaceutical interest.

## Supplementary Material

fiae117_Supplemental_Files

## Data Availability

This Whole Genome Shotgun project has been deposited at DDBJ/ENA/GenBank under the accession JBAIZT000000000. The version described in this paper is version JBAIZT010000000.1. The sequences are publicly available under BioProject accession number PRJNA1074715, BioSample accession number SAMN39880939, and SRA accession number SRR27945399.
