# Supplementary material for: Taxonomic and functional metagenomic assessment of a Dolichospermum bloom in a large and deep lake south of the Alps
Source: FEMS Microbiol Ecol. 2024 Sep 3;100(10):fiae117. doi: 10.1093/femsec/fiae117 (PMC11412076; doi:10.1093/femsec/fiae117)
Supplement: fiae117_Supplemental_Files [file fiae117_supplemental_files.zip › MAG_Dolichospermum_bloom_Suppl_Figures.pdf]

Taxonomic and functional metagenomic assessment of a *Dolichospermum* bloom in a large and deep lake south of the Alps

Nico Salmaso, Leonardo Cerasino, Massimo Pindo, Adriano Boscaini

Supplementary Figures

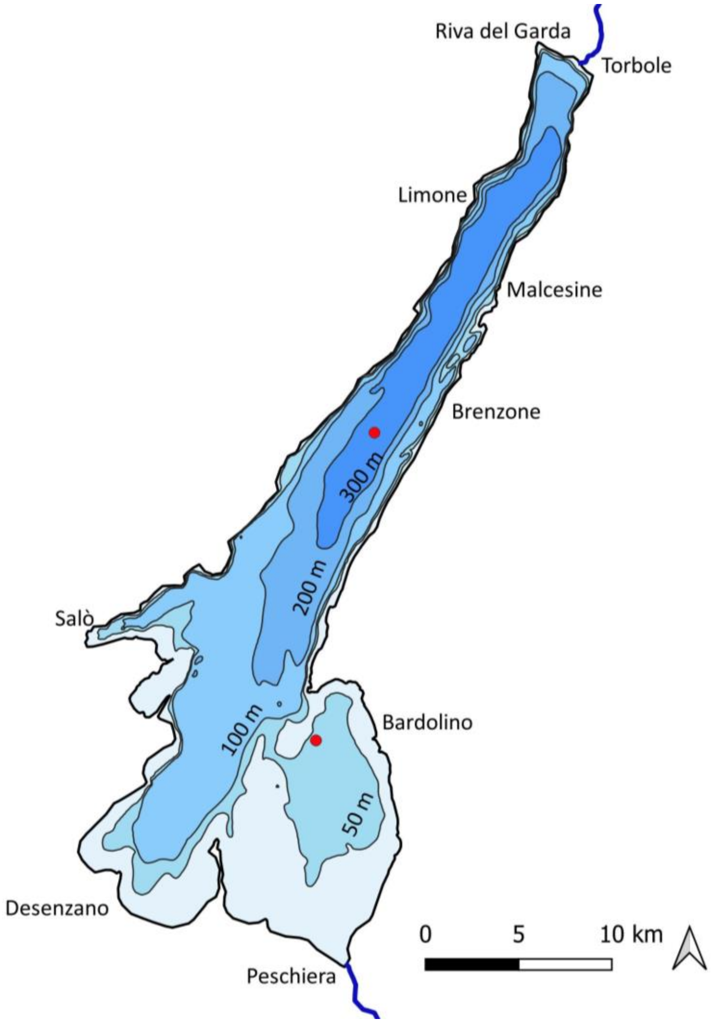

Supplementary Fig. 1. Map of Lake Garda. The sampling sites in the southeastern (Bardolino station) and northeastern (Brenzone station) basins are marked with red circles. The bloom was observed in the southeastern basin, outside the town of Bardolino.

(A) (B)

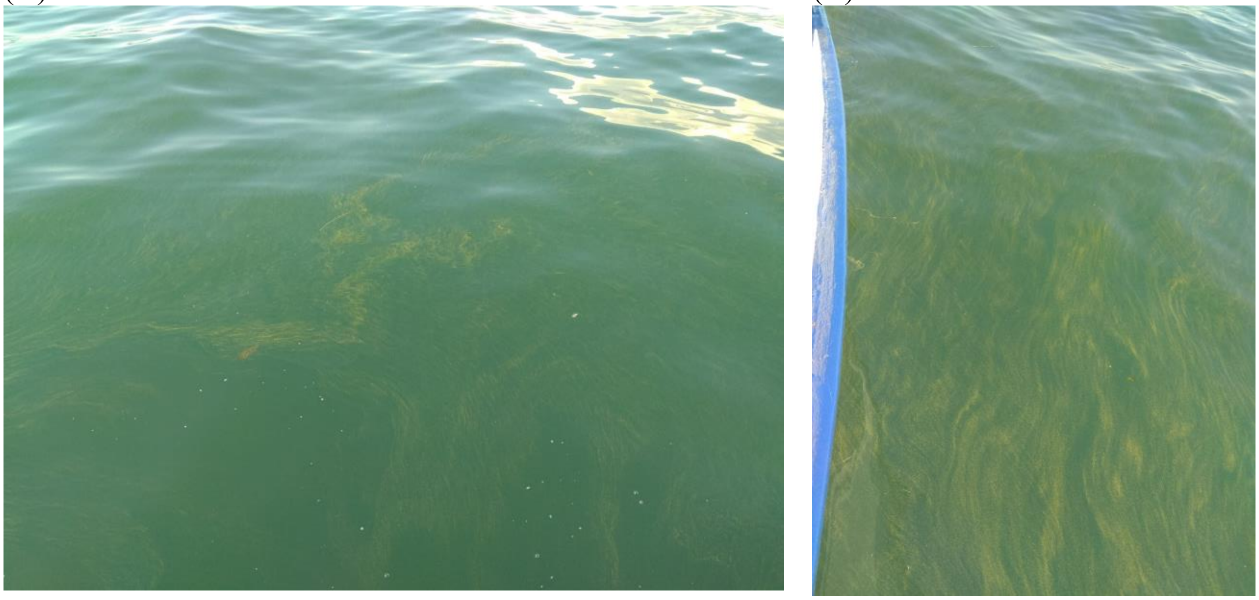

Supplementary Fig. 2. Oligotrophic surface bloom of *Dolichospermum lemmermannii* recorded in the southeastern basin of Lake Garda. Photos taken by Giorgio Franzini (ARPAV Verona) from the boat used for sampling on September 1, 2020.

(A) (B)

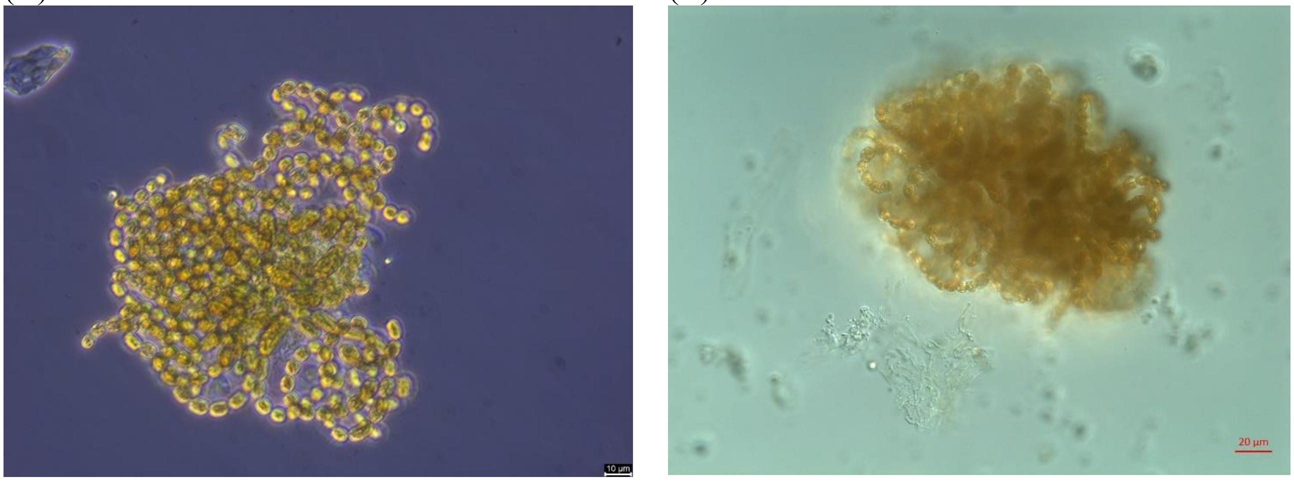

Supplementary Fig. 3. Coiled filaments of *Dolichospermum lemmermannii* collected on September 1, 2020, in the layer 0-1 m in the northeastern basin of Lake Garda. (A) Upright microscope, photo by A. Boscaini (FEM). (B) Inverted microscope, photo by A. Boscaini (FEM) and S. Pozzi (Provincial Environmental Protection Agency, Trento). Sample fixed in Lugol's solution.

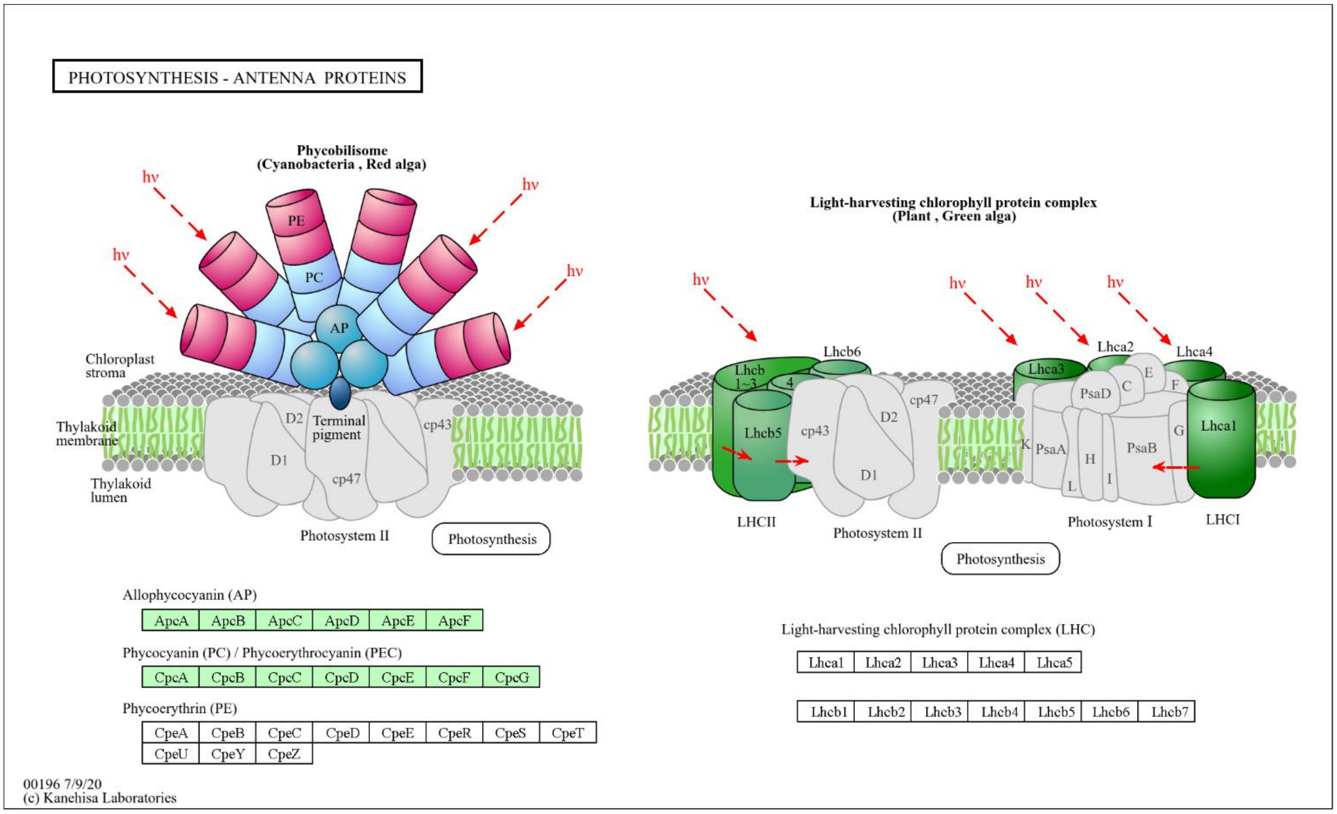

Supplementary Fig. 4. KEGG molecular pathways of photosynthetic antenna proteins identified by KEGG mapper (www.genome.jp/kegg/mapper) (Kanehisa and Sato 2020). Proteins corresponding to KEGG orthologies (KO) identified by GhostKOALA in the genome of *D. lemmermannii* FEM_B0920 are highlighted in green. Detailed and reference-based complete descriptions of the individual proteins are reported in https://www.kegg.jp/pathway/map00196.

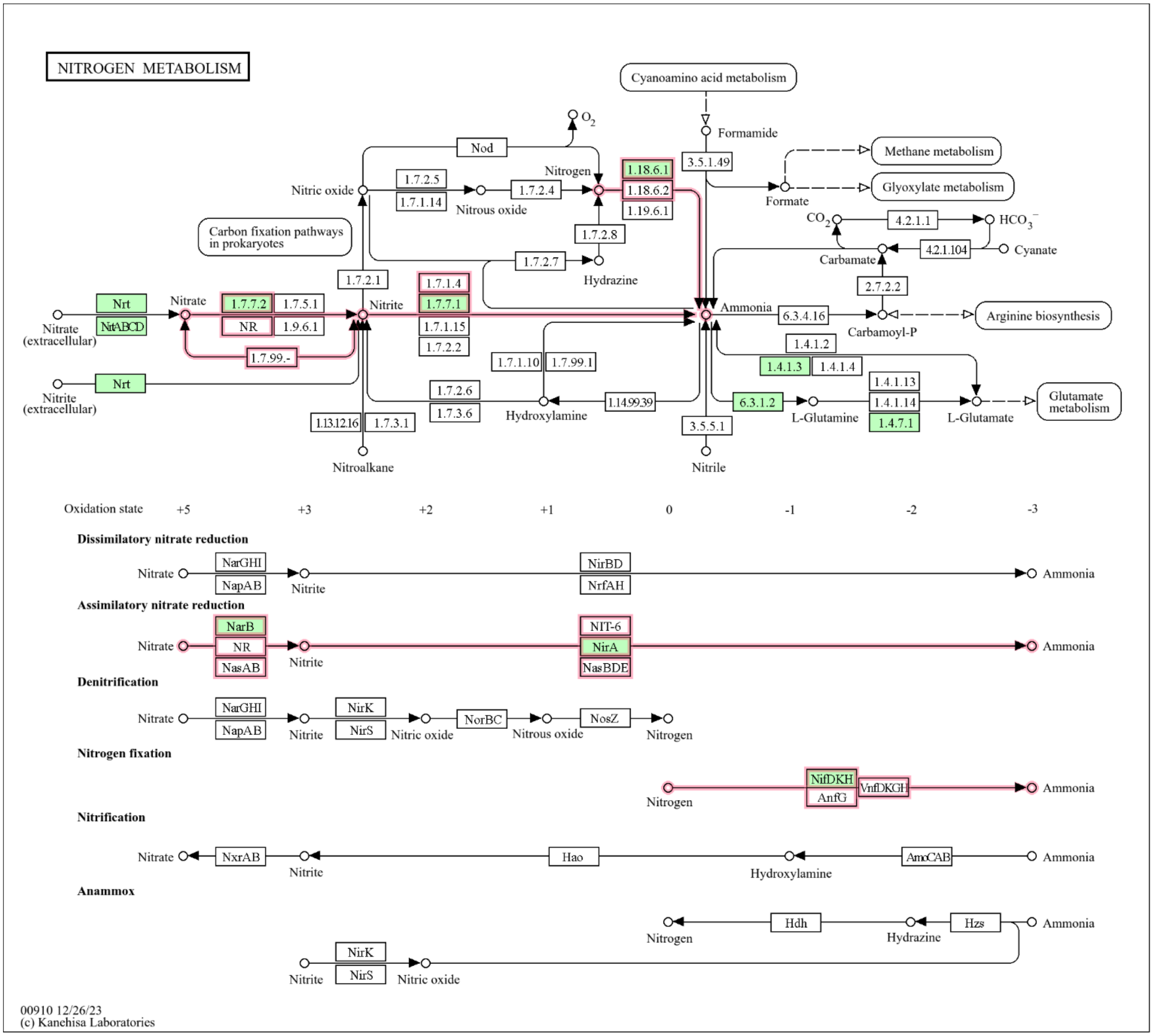

Supplementary Fig. 5. KEGG molecular pathways of nitrogen metabolism proteins identified by KEGG mapper (www.genome.jp/kegg/mapper) (Kanehisa and Sato 2020). Proteins corresponding to KEGG orthologies (KO) identified by GhostKOALA in the genome of *D. lemmermannii* FEM_B0920 are highlighted in green. Two main pathways modules have been identified, corresponding to M00531 (Assimilatory nitrate reduction) and M00175 (Nitrogen fixation). Detailed and reference-based complete descriptions of the individual proteins are reported in https://www.kegg.jp/pathway/map00910.

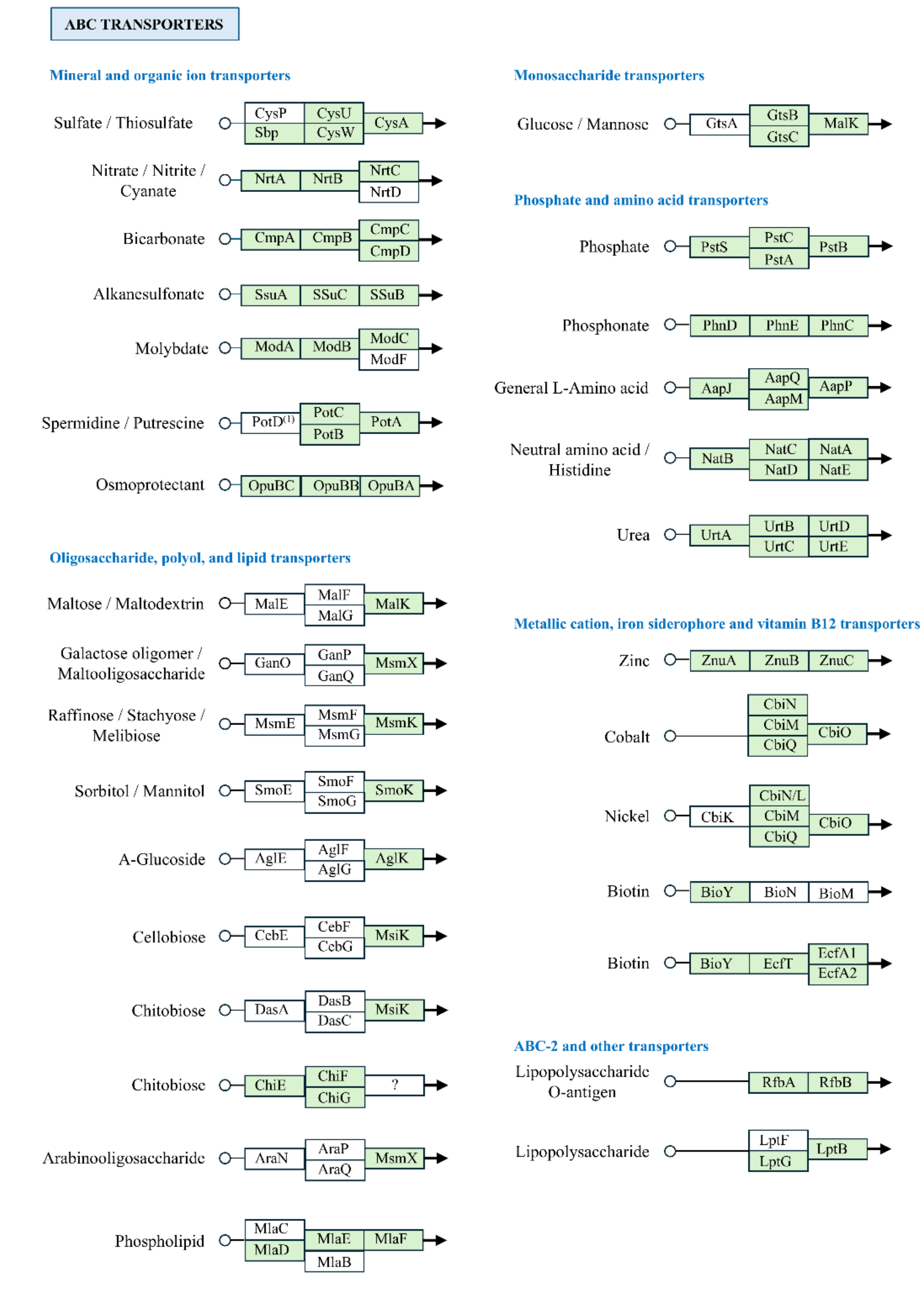

Redrawn from 02010 9/14/23 (02010 ABC Transporters) (c) Kanehisa Laboratories

Supplementary Fig. 6. Protein components of ATP-binding cassette (ABC) prokaryotic transporters (redrawn from 02010 9/14/23 (02010 ABC Transporters) (c) Kanehisa Laboratories). Proteins corresponding to KEGG orthologies identified by GhostKOALA and KEGG mapper in the genome of *D. lemmermannii* FEM_B0920 are highlighted in green. Except for a protein part of the spermidine/putrescine not identified in the Garda MAG[(1)], identical results were obtained for *D. lemmermannii* CS-548. ABC classifications based on Igarashi *et al.* (2004) and Tomii and Kanehisa (1998). Detailed and reference-based complete descriptions of the individual proteins are reported in https://www.kegg.jp/pathway/map02010.

References

Igarashi Y, Aoki KF, Mamitsuka H *et al.* The evolutionary repertoires of the eukaryotic-type ABC transporters in terms of the phylogeny of ATP-binding domains in eukaryotes and prokaryotes. *Mol Biol Evol* 2004;**21**:2149–60.

Kanehisa M, Sato Y. KEGG Mapper for inferring cellular functions from protein sequences. *Protein Sci* 2020;**29**:28–35.

Tomii K, Kanehisa M. A comparative analysis of ABC transporters in complete microbial genomes. *Genome Res* 1998;**8**:1048–59.
