# Supplementary material for: Taxonomic and functional metagenomic assessment of a Dolichospermum bloom in a large and deep lake south of the Alps
Source: FEMS Microbiol Ecol. 2024 Sep 3;100(10):fiae117. doi: 10.1093/femsec/fiae117 (PMC11412076; doi:10.1093/femsec/fiae117)
Supplement: fiae117_Supplemental_Files [file fiae117_supplemental_files.zip › MAG_Dolichospermum_bloom_Suppl_Table_1.pdf]

Taxonomic and functional metagenomic assessment of a *Dolichospermum* bloom in a large and deep lake south of the Alps

Nico Salmaso, Leonardo Cerasino, Massimo Pindo, Adriano Boscaini

Supplementary Table 1

Supplementary Table 1. Genera belonging or potentially belonging to the ADA group (*Anabaena*, *Dolichospermum* and *Aphanizomenon*) included in the phylogenomic analyses of Figures 1 and 2. NCBI assembly codes: GenBank assembly accession codes; NCBI organism name: the taxon name adopted in the NCBI GenBank taxonomy; GTDB organism name: the classification adopted in the GTDB (Genome Taxonomy Database) taxonomy; the *Dolichospermum* identified in the bloom of Lake Garda has been highlighted in bold. ADA: species previously included in the ADA categories by Driscoll et al. (2018) and Dreher, Davis and Mueller (2021); ADA+: assignment of ADA tags based on membership in clades defined in the phylogenomic tree of Fig. 2. The last six columns report the similarity of the identified BGCs to known clusters of genes encoding MC (microcystin), ATX (anatoxin), STX (saxitoxin/gonyautoxin and analogues), CYN (cylindrospermopsin), APs (anabaenopeptin and anabaenopeptin/nostamide), and GEO (geosmin). Low similarity values have to be interpreted with great care; with a few exceptions (see Notes), the results for MC, ATX, STX, CYN and GEO obtained with antismash have been supported by the corresponding genes detected by ISeqDb. In ADA-1 (*D. circinale*), a few CS- strains were reported in ANACC (2022) with a “Positive” toxicity (†), but without reporting a specific toxicity description; for this group of genomes, in the STX column, § and ¥ indicate the detection of saxitoxin with analytical methods available only for the two reported strains (Beers 2020) and detection of the *sxtA* gene (pident 100%, 1242 bp) by IseqDb, respectively.

NCBI assembly code	NCBI Organism Name	GTDB Organism name	Country	ADA	ADA+	MC	ATX	STX	CYN	APs	GEO
GCA_021300315.1	Anabaena sp. 49628 E55	Dolichospermum circinale	USA		ADA-1						
GCA_000426905.1	Dolichospermum circinale AWQC131C	Dolichospermum circinale	AUS	ADA-1	ADA-1			78			100
GCA_000426925.1	Dolichospermum circinale AWQC310F	Dolichospermum circinale	AUS	ADA-1	ADA-1						100
GCA_028331265.1	Dolichospermum circinale CS-1031 †	Dolichospermum circinale	AUS		ADA-1			§ ¥			100
GCA_028330925.1	Dolichospermum circinale CS-534/05	Dolichospermum circinale	AUS		ADA-1						100
GCA_028330865.1	Dolichospermum circinale CS-537/01 †	Dolichospermum circinale	AUS		ADA-1						100
GCA_028330805.1	Dolichospermum circinale CS-537/03 †	Dolichospermum circinale	AUS		ADA-1			¥			100
GCA_028330295.1	Dolichospermum circinale CS-537/11 †	Dolichospermum circinale	AUS		ADA-1			¥			100
GCA_028329385.1	Dolichospermum circinale CS-539	Dolichospermum circinale	AUS		ADA-1						100
GCA_028330025.1	Dolichospermum circinale CS-539/09	Dolichospermum circinale	AUS		ADA-1						100
GCA_028328745.1	Dolichospermum circinale CS-541/04 †	Dolichospermum circinale	AUS		ADA-1			¥			100
GCA_028331065.1	Dolichospermum circinale CS-541/06 †	Dolichospermum circinale	AUS		ADA-1			§ ¥			100
GCA_014696335.1	Anabaena sp. FACHB-1250	Dolichospermum planctonicum	CHN		ADA-1						100
GCA_014696755.1	Anabaena sp. FACHB-1391	Dolichospermum planctonicum	CHN		ADA-1						100
GCA_015207785.1	Dolichospermum flos-aquae LEGE 04289[4]	Dolichospermum planctonicum	PRT		ADA-1						
GCA_028330995.1	Dolichospermum planctonicum CS-1226	Dolichospermum planctonicum	AUS		ADA-1						100
GCA_005402965.1	Dolichospermum planctonicum NIES-80	Dolichospermum planctonicum	JPN	ADA-1	ADA-1						
GCA_009712075.2	Dolichospermum planctonicum UHCC 0167	Dolichospermum planctonicum	FIN	ADA-1	ADA-1						
GCA_001672075.1	Anabaena sp. CRKS33	Dolichospermum sp001672075	USA	ADA-1	ADA-1						100
GCA_000312705.1	Anabaena sp. 90	Dolichospermum sp000312705	FIN	ADA-2	ADA-2	100					
GCA_001672255.1	Anabaena sp. AL09[5]	Dolichospermum sp000312705		ADA-2						100	
GCA_001672225.1	Anabaena sp. LE011-02	Dolichospermum sp000312705		ADA-2						100	
GCA_028329755.1	Dolichospermum circinale CS-547[6]	Dolichospermum sp000312705	NOR		ADA-2	23[2]					
GCA_012516395.1	Dolichospermum flos-aquae CCAP 1403/13F	Dolichospermum sp000312705	GBR	ADA-2	ADA-2					88	
GCA_028330815.1	Dolichospermum lemmermannii CS-548	Dolichospermum sp000312705	NOR		ADA-2	76				100	
GCA_024998765.1	Dolichospermum sp.	Dolichospermum sp000312705	USA		ADA-2	92				100	
GCA_018447705.1	Dolichospermum sp. BR01	Dolichospermum sp000312705	USA	ADA-2	ADA-2					100	100
GCA_017355625.1	Dolichospermum sp. DET73	Dolichospermum sp000312705	USA	ADA-2	ADA-2	100				100	

GCA_017355645.1	Dolichospermum sp. DEX182a	Dolichospermum sp000312705	USA	ADA-2	ADA-2						
GCA_017346835.1	Dolichospermum sp. DL01	Dolichospermum sp000312705	USA	ADA-2	ADA-2						
GCA_017355635.1	Dolichospermum sp. JUN01	Dolichospermum sp000312705	USA	ADA-2	ADA-2	92				88	
GCA_017346815.1	Dolichospermum sp. LBC05a	Dolichospermum sp000312705	USA	ADA-2	ADA-2	92				100	
GCA_018447755.1	Dolichospermum sp. OL01	Dolichospermum sp000312705	USA	ADA-2	ADA-2	92				100	
GCA_024036375.1	Dolichospermum sp. OL03	Dolichospermum sp000312705	USA		ADA-2	92				100	
GCA_009711985.1	Dolichospermum sp. UHCC 0260	Dolichospermum sp000312705	FIN	ADA-2	ADA-2	76				100	
GCA_009711965.1	Dolichospermum sp. UHCC 0299	Dolichospermum sp000312705	FIN	ADA-2	ADA-2					100	
GCA_008121535.1	Dolichospermum sp. UHCC 0315A	Dolichospermum sp000312705	FIN	ADA-2	ADA-2	84				100	
GCA_009711925.1	Dolichospermum sp. UHCC 0352	Dolichospermum sp000312705	FIN	ADA-2	ADA-2	92				66	
GCA_009712025.1	Dolichospermum sp. UHCC 0406	Dolichospermum sp000312705	FIN	ADA-2	ADA-2					100	
GCA_017346795.1	Dolichospermum sp. UKL201	Dolichospermum sp000312705	USA	ADA-2	ADA-2					100	
GCA_018447775.1	Dolichospermum sp. WA123	Dolichospermum sp000312705	USA	ADA-2	ADA-2					100	
**GCA_037075685.1**	**Dolichospermum lemmermannii FEM B0920**	**NA**	**ITA**	**ADA-2**	**ADA-2**					**44**	**100**
GCA_017355725.1	Anabaena sp. 54	Dolichospermum heterosporum	FIN	ADA-3	ADA-3		53				
GCA_001672085.1	Anabaena sp. AL93	Dolichospermum heterosporum	USA		ADA-3		53				
GCA_001277295.1	Anabaena sp. WA102	Dolichospermum heterosporum	USA	ADA-3	ADA-3		53				
GCA_014696815.1	Aphanizomenon flos-aquae FACHB-1040	Dolichospermum heterosporum	CHN		ADA-3		53			100	
GCA_017346875.1	Aphanizomenon flos-aquae KM1D3_PB	Dolichospermum heterosporum	LTU	ADA-3	ADA-3						
GCA_000521175.1	Aphanizomenon flos-aquae NIES-81	Dolichospermum heterosporum	JPN	ADA-3	ADA-3					100	100
GCA_030130105.1	Aphanizomenon sp. PH219	Dolichospermum heterosporum	DNK		ADA-3					100	100
GCA_009712065.1	Aphanizomenon sp. UHCC 0183	Dolichospermum heterosporum	FIN	ADA-3	ADA-3						
GCA_009712125.1	Dolichospermum flos-aquae UHCC 0037	Dolichospermum heterosporum	FIN	ADA-3	ADA-3		53				
GCA_024584745.1	Dolichospermum heterosporum TAC447	Dolichospermum heterosporum	JPN		ADA-3					100	100
GCA_017355655.1	Dolichospermum sp. DEX189	Dolichospermum heterosporum	USA	ADA-3	ADA-3					100	100
GCA_015207865.1	Dolichospermum sp. LEGE 00246	Dolichospermum heterosporum	PRT		ADA-3					57	100
GCA_026389355.1	Nostocales cyanobacterium LacPavin_0920_SED1_MAG_38_18	Dolichospermum heterosporum	FRA		ADA-3						
GCA_021297915.1	Anabaena sp. CoA2_C59	Dolichospermum flosaquae	USA		ADA-4						
GCA_003525565.1	Anabaena sp. UBA12330	Dolichospermum flosaquae			ADA-4						
GCA_001672155.1	Anabaena sp. WA113	Dolichospermum flosaquae	USA	ADA-4	ADA-4						
GCA_014654815.1	Aphanizomenon flos-aquae Clear-A1	Dolichospermum flosaquae	USA		ADA-4						
GCA_017355665.1	Aphanizomenon flos-aquae CP01	Dolichospermum flosaquae	USA	ADA-4	ADA-4						
GCA_017346855.1	Aphanizomenon flos-aquae DEX188	Dolichospermum flosaquae	USA	ADA-4	ADA-4						
GCA_014697315.1	Aphanizomenon flos-aquae FACHB-1171	Dolichospermum flosaquae	CHN		ADA-4						
GCA_014698725.1	Aphanizomenon flos-aquae FACHB-1249	Dolichospermum flosaquae	CHN		ADA-4						
GCA_014698705.1	Aphanizomenon flos-aquae FACHB-1265	Dolichospermum flosaquae	CHN		ADA-4						
GCA_014698755.1	Aphanizomenon flos-aquae FACHB-1287	Dolichospermum flosaquae	CHN		ADA-4						
GCA_014698295.1	Aphanizomenon flos-aquae FACHB-1290	Dolichospermum flosaquae	CHN		ADA-4						
GCA_014698695.1	Aphanizomenon flos-aquae FACHB-1416	Dolichospermum flosaquae	CHN		ADA-4						
GCA_001672165.1	Aphanizomenon flos-aquae LD13	Dolichospermum flosaquae	USA	ADA-4	ADA-4						
GCA_001672095.1	Aphanizomenon flos-aquae MDT14a	Dolichospermum flosaquae	USA	ADA-4	ADA-4						
GCA_001593825.2	Aphanizomenon flos-aquae UKL13-PB	Dolichospermum flosaquae	USA		ADA-4						
GCA_001672105.1	Aphanizomenon flos-aquae WA102	Dolichospermum flosaquae	USA	ADA-4	ADA-4						
GCA_014698245.1	Aphanizomenon sp. FACHB-1399	Dolichospermum flosaquae	CHN		ADA-4						
GCA_014698265.1	Aphanizomenon sp. FACHB-1401	Dolichospermum flosaquae	CHN		ADA-4						
GCA_030065355.1	Nostocales cyanobacterium LE14-WE12	Dolichospermum flosaquae	USA		ADA-4						
GCA_009712035.1	Anabaena sp. UHCC 0187	Dolichospermum sp009712035	FIN	ADA-5	ADA-5						
GCA_018295975.1	Dolichospermum sp. DET50	Dolichospermum sp017355425	USA		ADA-6				48		
GCA_018295985.1	Dolichospermum sp. DET66	Dolichospermum sp017355425	USA		ADA-6				48		
GCA_018295965.1	Dolichospermum sp. DET67	Dolichospermum sp017355425	USA		ADA-6				48		

GCA_017355425.1	Dolichospermum sp. DET69	Dolichospermum sp017355425	USA	ADA-6	ADA-6				48		
GCA_009711975.1	Anabaena sp. UHCC 0204	Dolichospermum sp009711975	FIN	ADA-7	ADA-7						
GCA_009712085.1	Anabaena sp. UHCC 0253	Dolichospermum sp009711975	FIN	ADA-7	ADA-7						
GCA_002934005.1	Cuspidothrix issatschenkoi CHARLIE-1[(1)]	Cuspidothrix issatschenkoi	CZE	ADA-8	ADA-8		53				
GCA_009711935.1	Dolichospermum sp. UHCC 0259	Dolichospermum sp009711935	FIN	ADA-9	ADA-9						
GCA_002368115.1	Dolichospermum compactum NIES-806	Dolichospermum compactum		ADA-10	ADA-10					100	
GCA_030336445.1	Aphanizomenon gracile PMC627.10	Dolichospermum gracile	FRA					58	8[(3)]	50	
GCA_030336425.1	Aphanizomenon gracile PMC638.10	Dolichospermum gracile	FRA					58			
GCA_030336385.1	Aphanizomenon gracile PMC644.10	Dolichospermum gracile	FRA								
GCA_030336405.1	Aphanizomenon gracile PMC649.10[(7)]	Dolichospermum gracile	FRA						8[(3)]		
GCA_028330045.1	Aphanizomenon sp. CS-733/32	Dolichospermum gracile									
GCA_015207875.1	Dolichospermum sp. LEGE 00240[(4)]	Dolichospermum gracile	PRT					58		50	
GCA_014697585.1	Dolichospermum sp. FACHB-1091	Dolichospermum sp014697585	CHN								
GCA_028658625.1	Dolichospermum sp. ST_sed1	Dolichospermum sp028658405	DEU							55	
GCA_028658605.1	Dolichospermum sp. ST_sed2	Dolichospermum sp028658405	DEU							85	
GCA_028658555.1	Dolichospermum sp. ST_sed3	Dolichospermum sp028658405	DEU							55	
GCA_028658525.1	Dolichospermum sp. ST_sed4	Dolichospermum sp028658405	DEU							85	
GCA_028658575.1	Dolichospermum sp. ST_sed6	Dolichospermum sp028658405	DEU							85	
GCA_028658505.1	Dolichospermum sp. ST_sed7	Dolichospermum sp028658405	DEU							85	
GCA_028658405.1	Dolichospermum sp. ST_sed8	Dolichospermum sp028658405	DEU							85	

NOTES: [(1)]Outgroup in the trees of Figures 1 and 2. [(2)]*mcy* genes not identified by IseqDb. [(3)]*CyrJ* gene not identified by ISeqDb. [(4)]Reported as ATX-a producing strain by Ramos *et al.* (2018). [(5)]APs not indicated in Österholm *et al.* (2020). [(6)]"Toxicity" below detection limit (ANACC 2022). [(7)]*sxtA* identified using Sanger sequencing (Ledreux *et al.* 2010) in cultures analysed in 2010 (Halary *et al.* 2023).

References

ANACC. *Strain List 2022*. Australia's National Science Agency, 2022.

Beers EN. *Investigating the Effects of Temperature on the Growth and Toxin Production of Saxitoxin, Anatoxin and Cylindrospermopsin Producing Cyanobacteria - MSc Thesis*. Ohio: Bowling Green State University, 2020:1–30.

Dreher TW, Davis EW, Mueller RS. Complete genomes derived by directly sequencing freshwater bloom populations emphasize the significance of the genus level ADA clade within the Nostocales. *Harmful Algae* 2021;**103**:102005.

Driscoll CB, Meyer KA, Šulčius S *et al.* A closely-related clade of globally distributed bloom-forming cyanobacteria within the Nostocales. *Harmful Algae* 2018;**77**:93–107.

Halary S, Duperron S, Kim Tiam S *et al.* Intra-population genomic diversity of the bloom-forming cyanobacterium, *Aphanizomenon gracile*, at low spatial scale. *ISME Commun* 2023;**3**:57.

Ledreux A, Thomazeau S, Catherine A *et al.* Evidence for saxitoxins production by the cyanobacterium *Aphanizomenon gracile* in a French recreational water body. *Harmful Algae* 2010;**10**:88–97.

Österholm J, Popin RV, Fewer DP *et al.* Phylogenomic Analysis of Secondary Metabolism in the Toxic Cyanobacterial Genera Anabaena, Dolichospermum and Aphanizomenon. *Toxins* 2020;**12**:248.

Ramos V, Morais J, Castelo-Branco R *et al.* Cyanobacterial diversity held in microbial biological resource centers as a biotechnological asset: the case study of the newly established LEGE culture collection. *J Appl Phycol* 2018;**30**:1437–51.
