# Supplementary material for: Taxonomic and functional metagenomic assessment of a Dolichospermum bloom in a large and deep lake south of the Alps
Source: FEMS Microbiol Ecol. 2024 Sep 3;100(10):fiae117. doi: 10.1093/femsec/fiae117 (PMC11412076; doi:10.1093/femsec/fiae117)
Supplement: fiae117_Supplemental_Files [file fiae117_supplemental_files.zip › MAG_Dolichospermum_bloom_Suppl_Table_2.pdf]

Taxonomic and functional metagenomic assessment of a *Dolichospermum* bloom in a large and deep lake south of the Alps

Nico Salmaso, Leonardo Cerasino, Massimo Pindo, Adriano Boscaini

Supplementary Table 2

Supplementary Table 2. Genes of taxonomic relevance and involved in the biosynthesis of geosmin, obtained from *Dolichospermum lemmermannii* FEM_B0920.

>16S ribosomal RNA gene *Dolichospermum lemmermannii* FEM_B0920
AAAATGGAGAGTTTGATCCTGGCTCAGGATGAACGCTGGCGGTATGCTTAACACATGCAAGTCGAACGGAATCCTTAGGGATTTAGTGGCGGACGGGTGAGTA
ACGCGTAAGAATCTACCTTCAGGTTGGGGACAACCACTGGAAACGGTGGCTAATACCGAATGTGCCGAGAGGTGAAAGGCTTGCTGCCTGAAGAAGAGCTTGC
GTCTGATTAGCTAGTTGGTGTGGTAAAGGCGCACCAAGGCGACGATCAGTAGCTGGTCTGAGAGGATGATCAGCCACACTGGGACTGAGACACGGCCCAGACT
CCTACGGGAGGCAGCAGTGGGGAATTTTCCGCAATGGGCGAAAGCCTGACGGAGCAATACCGCGTGAGGGAGGAAGGCTCTTGGGTTGTAAACCTCTTTTCTC
AGGGAAGAAAAAAATGACGGTACCTGAGGAATAAGCATCGGCTAACTCCGTGCCAGCAGCCGCGGTAATACGGAGGATGCAAGCGTTATCCGGAATGATTGGG
CGTAAAGGGTCCGCAGGTGGCATTGTTTGTCTGCTGTTAAAGAGTTTGGCTCAACCAAATAAGAGCAGTGGAAACTACAAAGCTAGAGTATGGTCGGGGCAGA
GGGAATTCCTGGTGTAGCGGTGAAATGCGTAGATATCAGGAAGAACACCGGTGGCGAAGGCGCTCTGCTAGGCCAAAACTGACACTGAGGGACGAAAGCTAGG
GGAGCGAATGGGATTAGATACCCCAGTAGTCCTAGCCGTAAACGATGGATACTAGGCGTAGCTCGTATCGACCCGAGCTGTGCCGTAGCTAACGCGTTAAGTA
TCCCGCCTGGGGAGTACGCAGGCAACTGTGAAACTCAAAGGAATTGACGGGGGCCCGCACAAGCGGTGGAGTATGTGGTTTAATTCGATGCAACGCGAAGAAC
CTTACCAAGACTTGACATGTCACGAATCCCGGCGAAAGTTGGGAGTGCCTTAGGGAGCGTGAACACAGGTGGTGCATGGCTGTCGTCAGCTCGTGTCGTGAGA
TGTTGGGTTAAGTCCCGCAACGAGCGCAACCCTCGTTTTTAGTTGCCAGCATTAAGTTGGGCACTCTAGAGAGACTGCCGGTGACAAACCGGAGGAAGGTGGG
GATGACGTCAAGTCAGCATGCCCCTTACGTCTTGGGCTACACACGTACTACAATGCTCCGGACAAAGGGCAGCTACACAGCGATGTGATGCGAATCTCATAAA
CCGGAGCTCAGTTCAGATCGAAGGCTGCAACTCGCCTTCGTGAAGGAGGAATCGCTAGTAATTGCAGGTCAGCATACTGCAGTGAATTCGTTCCCGGGCCTTG
TACACACCGCCCGTCACACCATGGAAGTTGGTCACGCCCGAAGTCGTTACCCCAACCTTTTGGAGGGGGATGCCTAAGGTAGGACTGATGACTGGGGTGAAGT
CGTAACAAGGTAGCCGTACCGGAAGGTGTGGCTGGATCACCTCCTTT

>23S ribosomal RNA gene *Dolichospermum lemmermannii* FEM_B0920
GGTCAAGTTAATAAGGGCTAATGGTGGATACCTAGGCACACAGAGGCGATGAAGGACGTGGTTACCGACGAAATGTTCCGGGGAGTTGGAAGCAAACTTTGAG
CCGGAAATGTCCGAATGGGGCAACCCTATGTACTACCTGTTGAATATATAGACAGGAAAGAGCCAACCCAGCGAACTGAAACATCTTAGTAGCTGGAGGAAGA
GAAATCAAACTAGAGATTCCCTCAGTAGTGGTGAGCGAAAGGGGAAGAGCCTAAACCAAGGGATTTATCCTTTGGGGTCGTGGGACAGCAATATCGAATCTAG
AGGTTAGACGAAACAGCTAAATACTGTACCAGAGGAAGTGAAAGTCTTGTAGTCAAAAACCAAAGGATAGTAGCTGAATCCCGAGTAGCATGGAGCACGAGGA
ATTCCATGTGAATCAGCGAGGACCACCTCGTAAGGCTAAATACTACTGTGTGACCGATAGTGAACAAGTACCGCGAGGGAAAGGTGAAAAGAACCCCGGAAGG
GGAGTGAAATAGAACATGAAACCATAAGCTTACAAGCAGTGGGAGTCCGATTTAACGGATGACCGCGTGCCTGTTGAAGAATGAGCCGGCGACTTATAGGCAC
TGGTAGGTTAAAGCGAGAATGCTGGAGCCAAAGGGAAACCGAGTCTGAACAGGGCGATAAATCAGTGTTTATAGACCCGAACCCTGGTGATCTAACCATGTCC
AGGATGAAGCTTGGGTAACACCAAGTGGAGGTCCGAACCGACTGATGTTGAAAAATCAGCGGATGAGGTGTGGTTAGGGGTGAAATACCAATCGAACCAGGAG
CTAGCTGGTTCTCCCCGAAATGTGTTGAGGCGCAGCGGTAATGGTTAAATTTGGGGGGTAAAGCACTGTTTCGGTGCGGGCTGGGAGACCGGTACCAAATCGA
GACAAACTCAGAATACCCAGAAGAAGACATTGCCAGTGAGACAGTGGGGGATAAGCTTCATTGTCAAGAGGGAAACAGCCCAGACCACCAGCTAAGGTCCCCA
AATCATCGCTAAGTGATAAAGGAGGTGAGATTGCCTAGACAACTAGGAGGTTTGCCTAGAAGCAGCCACCCTTGAAAGAGTGCGTAATAGCTCACTAGTCAAG
CGATCTTGCGCCGAAAATGAACGGGGCTAAGCGATGTACCGAAGCTGTGGGATTGATATTAAATTATTAATCGGTAGGGGAGCGTTCCGTCGTAGGTTGAAGC
AGTAGCGGCGAGCAGCTGTGGACGAAACGGAAGTGAGAATGTCGGCTTGAGTAGCGCAAATGTATGTGAGAATCATACACCCCGAAACCCTAAGGGTTCCAGA
GCCAGGTTCGTCCACTCTGGGTTAGTCGGGACCTAAGGCGAGGCCGAAGGGCGTAGTCGATGGACAAAGTGTCAATAGTCACTTACTGTTCTGTGGGAGCATA
GAGAGGGACGCATGAAAGATAGCCATACCCTGATTGGTTTGGGAGGAGTTTACGAACTCCGAGTGGTGAAGGATAGTGCCAAGAAAAGCTGGCTATGTGATGA
AGATAGAACACCCGTACCCGAAACCGACACAGGTAGGGAGGTTGAGAATACCAAGGGGCGCGAGATAACTCTCTCTAAGGAACTCGGCAAAATGGCCCCGTAA
CTTCGGGAGAAGGGGTGCCCACCTCAGACGTGGGTCGCAGTGAAGAGATCCAGGCGACTGTTTACCAAAAACACAGGTCTCCGCCAACTCGCAAGAGGACGTA
TGGGGGCTGACGCCTGCCCAGTGCCGGAAGGTTAAGGAAGCTGGTCAGCGAAAGTAAAGCTGGCGACTGAAGCCCCGGTGAACGGCGGCCGTAACTATAACGG
TCCTAAGGTAGCGAAATTCCTTGTCGGGTAAGTTCCGACCCGCACGAAAGGCGTAACGATCTGGATGGTGTCTCAGAGAGAGACTCGGCGAAATAGGAATGTC
TGTGAAGATACGGACTGCCTGCACCTGGACAGAAAGACCCTATGAAGCTTTACTGTAGCCTGGAATTGTGTTCGGGCTTCGCTTGCGCAGGATAGGTGGGAAG
CGGTGAACTATTCCTTTTGGGGAATAGGGAGCTAACGGTGAGATACCACTCTGGCGAAGCTAGAATTCTAACCCACGACCGTCAGCCGGTTGGGGAACAGTTT
CAGGTGGGCAGTTTGACTGGGGCGGTCGCCTCCTAAAAGGTAACGGAGGCGCGCAAAGGTTCCCTCAGCACGCTTGGAAACCGTGCGACGAGTGTAAAAGCAA
AAGGGAGCTTGACTGCAAGACTGACAAGTCGAGCAGGTACGAAAGTAGGCTTTAGTGATCCGACGGCGCAGAGTGGAATGGCCGTCGCTCAACGGATAAAAGT
TACTCTAGGGATAACAGGCTGATCTCCCCCAAGAGTCCACATCGACGGGGAGGTTTGGCACCTCGATGTCGGCTCATCGCAACCTGGGGCGGAAGTACGTCCC
AAGGGTTGGGCTGTTCGCCCATTAAAGCGGTACGTGAGCTGGGTTCAGAACGTCGTGAGACAGTTCGGTCCATATCCGGTGCAGGCGCAAGAGTATTGAGAGG
AGTCCTCCTTAGTACGAGAGGACCGGGAGGAACGCACCGCTGGTGTACCAGTTATTGTACCAACAGTAAACGCTGGGTAGCCAAGTGCGGAGAGGATAACCGC
TGAAAGCATCTAAGTGGGAAGCCCACCTCAAGATGAGTACTCTCACTACAAAAAGTAGGTAAGGTCACGGGCAGAACACCCGTTAATAGGCTCTAAGTGGAAG
TACAGTAATGTATGAAGCTGAGGGGTACTAATAGACCGAGGGCTTGACCTC

>rbcX ribulose bisphosphate carboxylase *Dolichospermum lemmermannii* FEM_B0920
ATGGATTTAAAGCAAATTGCGAAAGACACAGCCAAAACTCTCCAAAGCTACCTGACTTATCAGGCGTTGAGGACTGTATTGGCACAGCTAGGCGAAACAAATC
CTCCTCTAGCACATTGGTTGCAAAACTTCTCCGCTGGCAAAATCCAAGACGGAGAAGCATATATTGAGGAACTGTTTCTAGAAAAGTCAGATTTGGCTTTGCG
GATTATGACTGTCAGGGAACACATAGCGGCGGAAGTGACAGAGTTTTTACCAGAAATGGTGCTAACTGGCATTCAGCAAGCCAATATGGAACAGCGTCGCCAG
CATCTCGAACGCATTACGCAACTAAGTTTATCAAGTCCCAGCCTCGAAATACCAAAGACAGACAATATCTGATAGCCTGCGTGGCGTAGCTATTGCTAATTTG
GATAATTTATCCAATTAG

>rpoB RNA polymerase subunit beta *Dolichospermum lemmermannii* FEM_B0920
ATGACTAACGACGATAAATACATGGAATCCGCCTTTCTATTACCCGACTTGATTGAAATTCAGCGTTCTAGTTTTCGCTGGTTTCTAGAAGAAGGGCTAATCG
AAGAACTGAACTCCTTTAGTCCAATTACAGACTACACTGGCAAACTAGAACTGCACTTTTTAGGCAATAACTATAAACTCAAAGAACCTAAATACAGTGTTGA
AGAATCCAAGCGGAGAGATAGCACTTATGCAGTGCAAATGTATGTCCCCACCCGGTTGCTGAACAAAGAAACAGGAGACATTAAAGAACAAGAAGTATTTATT
GGCGATTTGCCATTGATGACAGATAGAGGCACATTTATTATCAACGGTGCTGAACGCGTCATTGTCAATCAAATTGTGCGATCGCCTGGAGTTTACTATAAAT
CAGAAATTGATAAAAACGGACGACGGACTTATTCTGCCAGCTTAATTCCGAACCGGGGGGCATGGCTGAAATTTGAAACAGATCGTAATGACTTAGTTTGGGT
ACGGATAGACAAAACCCGGAAACTGTCAGCCCAGGTACTGTTGAAAGCCCTGGGATTATCAGATAACGAGATATTTGATGCACTGCGCCATCCAGAATACTTC
CAAAAAACCATCGAAAAAGAAGGGCAATTTTCCGAAGAAGAAGCCCTAATGGAATTATATCGGAAACTTCGTCCCGGTGAACCGCCCACAGTTTTAGGTGGAC
AACAGCTTTTAGACTCCCGCTTCTTTGATCCTAAACGCTATGACCTGGGTAAAGTTGGACGTTATAAACTCAACAAAAAACTTCGTCTTTCCGCCCCCGACAC
CATGCGCGTCCTCACCCCTGGGGACATCTTAGCCGCCATTGATTACCTCATAAATCTAGAATATGACATCGGTAGTATTGATGACATTGACCACCTCGGAAAT

CGCCGAGTCAGAAGCGTTGGGGAATTGCTGCAAAACCAGGTCAGAGTAGGGTTAAATCGCTTAGAAAGAATTATTCGAGAACGGATGACCGTATCCGATGCCG
AATCTCTCACTCCCGCATCTCTAGTTAACCCCAAACCCTTAGTAGCAGCTATCAAAGAATTCTTTGGTTCGAGCCAACTCAGTCAATTCATGGATCAAACTAA
TCCCTTGGCAGAATTGACCCATAAACGCCGCCTCAGTGCCTTGGGACCAGGTGGTTTGACCAGAGAAAGAGCCGGGTTTGCAGTTCGAGATATTCACCCCAGC
CACTACGGACGGATTTGCCCCATTGAAACACCAGAAGGACCAAATGCCGGACTCATCGGTTCATTAGCCACCCATGCCCGCGTTAACCTCTATGGTTTTCTAG
AAACACCCTTCCGACCAGTAGAAAATGGCAAAGTCCTCTTTGATGTGCTGCCTGTTTACATGACAGCAGACGAAGAAGACGACCTGCGGACAGCCACAGGTGA
CGTTCCCCTCGATGAAAATGGTTACATCAAAGGACCCCACGTGCCAGTGCGCTATCGCCAAGACTGGACAACCACCGGACCCGAACAGGTGGATTATGTGGCA
GTTTCTCCAGTGCAGATTGTCTCAGTAGCCACCAGCATGATTCCCTTCTTGGAACATGACGACGCTAACCGAGCGCTCATGGGTTCTAATATGCAGCGCCAAG
CTGTACCCCTACTCAGACCAGAACGCCCCTTGGTGGGAACCGGTTTGGAAGCCCAAGCCGCCAGAGACTCCGGCATGGTGATAGTTTCCCGCACCGATGGCGA
TGTAGTCTATGTTGATGCCACAGAAATTCGTGTCCGCGCCAGTGGTCAGTTGTCCGCAGCCAGTGGTAGCCAAGTTATTGAAAAAGGCCAAGAACTTAAATAC
AAACTTTCCAAATATCAACGTTCCAACCAAGACACCTGTTTAAACCAAAAACCATTGGTGAGAATTGGCGAAAAAGTCGTAGCCGGTCAAGTATTGGCAGATG
GTTCTTCCACAGAAGGGGGAGAACTGGCGCTAGGACAAAATATAGTTGTCGCCTATATGCCCTGGGAAGGCTACAACTACGAGGACGCAATCCTCATTTCCGA
GCGCCTGGTACAGGAAGATGTCTATACTTCCATTCACATCGAAAAATACGAAATCGAAGCCAGACAAACCAAACTCGGACCCGAAGAAATAACCAGAGAAATT
CCCAACGTTGGTGAAGATGCCCTCAGACAATTGGATGAACAAGGCATTATCCGCATTGGGGCGTGGGTAGATGCTGGGGATATTCTCGTGGGAAAAGTTACTC
CCAAGGGTGAATCTGACCAACCCCCCGAAGAAAAACTCCTCCGCGCCATCTTTGGTGAAAAAGCCCGTGATGTCCGCGACAACTCCCTGCGAGTTCCCAATGG
TGAAAAAGGTCGGGTAGTTGATGTCCGGTTGTTCACCCGTGAACAAGGTGATGAACTGCCACCTGGGGCAAATATGGTAGTCCGGGTTTATGTGGCTCAAAAG
CGCAAAATCCAAGTGGGCGACAAAATGGCAGGGAGACATGGCAACAAGGGGATTATTTCCCGGATTCTGGCTGCTGAAGATATGCCCTACTTAGCTGACGGTT
CACCCGTGGATATTGTTCTCAATCCCTTGGGTGTACCCAGCCGGATGAATGTGGGTCAGGTGTTTGAATGTTTACTGGGTTGGGCAGGTCATAATTTGGGAGT
CAGGTTCAAGATTACTCCCTTCGATGAAATGTATGGCGAAGAAACTTCCCGGCGGCTAGTTCACGGCAAACTCCAAGAAGCCAGAGACGAAACCACTAAAGAC
TGGATCTATAACCCCGACAATGCCGGTAAAATCATGGTCTATGATGGTCGCACTGGTGAACCCTTTGACCGGGCTATTACCGTCGGTGTGGCCTATATGCTCA
AGCTCGTGCATTTGGTTGATGACAAAATTCACGCCCGTTCCACTGGACCCTATTCTCTGGTGACTCAGCAACCTTTGGGCGGTAAGGCTCAACAGGGTGGGCA
AAGATTTGGAGAAATGGAAGTTTGGGCGTTGGAAGCTTTCGGCGCTGCTTATACTTTGCAAGAATTGTTAACTGTCAAATCCGATGATATGCAAGGACGGAAT
GAAGCCCTCAATGCCATTGTCAAGGGTAAGGCTATTCCCCGTCCTGGTACTCCTGAATCTTTCAAGGTCTTAATGCGCGAGTTGCAATCTTTGGGTTTGGATA
TTGCGGTACATAAGGTGGAAACCCAAGCAGATGGTAGTTCCCTGGATGTGGAAGTAGACCTAATGGCAGACCAAGCTGCCCGGCGCACACCTCCTAGACCGAC
TTATGAGTCACTCTCTCGTGAGTCTCTGGAAGGGGAAGAGTAA

>Putative geosmin synthase (geo) gene *Dolichospermum lemmermannii* FEM_B0920
ATGCAACCTTTTAAACTCCCAGATTTTTATATGCCTTGGCCAGCTAGGCTGAATCCAAATCTGGAAGCAGCACGAGTGCATTCTAAGGCGTGGGCTTACGAAA
TGGGGATACTTGGCTCAAAAGAGGAAGCCCAAGGTGAGCCTATTTGGGACGAGCGTAAATTCGATGCTCATGACTACGCCTTGCTTTGCTCATATACCCATCC
AGACACAGATTCTCCAGAACTTGACTTGGTAACAGACTGGTATGTGTGGGTATTCTTTTTCGATGATCACTTCCTGGAAATCTATAAACGCAGTCAGGATATG
GTTGGGGCGAAGGAGTATCTTGACCGACTCCCCGCATTTATGCCGATTTATCCCAAGGGCAACCCTCCCGTTCCCACCAACCCAGTAGAGCGCGGTTTGGCTG
ACTTGTGGTCTCGCACCGCATTTACTAAGTCTGTGGAATGGCGGCGACGATTCTTTGAAAGTACCAAAAATCTTTTAGATGAGTCAATGTGGGAACTGGCCAA
CATCAATCAAAATCGAATTGCTAACCCCATCGAATACATTGAGATGCGGCGTAAAGTTGGTGGCGCACCCTGGTCAGCCGATCTGGTGGAACACGCCGCGTTT
GTGGAAGTTCCGGCTAAAATTGCGGCAACTAGGCCAATGCGGGTTTTAAAAGACACATTTGCTGATGGCGTACATCTCCGCAATGACCTATTCTCCTATCAAA
GAGAAGTGGAAGATGAAGGTGAAAATTCTAATTGTGTGCTTGTAATTGAAAAATTTTTGAATGTAAGTACCCAAGAGGCGGCTAACCTCACTAACGAACTACT
CAACTCCCGTTTATATCAGTTTGATAACACTGCTGTCACCGAGTTGCCCTCCCTTTTTGAGGAGTATGGAGTAGACCCAGTAGAGCGTGTGAATGTTCTCCTT
TACATCAAAGGACTTCAGGATTGGCAATCTGGCGGTCATGAGTGGCACATGAGATCAAGCCGCTATATGAATAAACAGGAAGAGGATAATTCTGCCAAATCTG
GTATTCTGAGTGGTCCAAATGGTTTAGGCACATCGGCAGCGCGGATTGAATCGTTATATACCACCTTGGGTTTAGGAAGGTTTAAAAGTTTTACTCACGTTCC
CTACCAGAATGTAGGTCCAGTGAAACTGCCGAAGTTTTATATGCCTTTCTCTACCACTTTGAATCCCAATCTAGATGCCGCGCGGAAGCATTCTAAGGAATGG
GCGCGTCAGATGGAGATGTTGGCATCACTACCTGGTATTCCTGATGCTTTTATCTGGAATGACCATAAGTTTGATGTTGCTGACGTAGCTTTATGCGGTGCGT
GGATACATCCAAAGGGTTCTGATCATGAGCTAAATTTAACAGCTTGCTGGCTTGTTTGGGGAACTTATGCTGATGATTATTTCCCCGCAATCTACGGGAATAA
CCGCGACTTAGCAGGTGCAAAAGTCTTTAACGCCCGACTGTCAGCGTTTATGCCTCTCGACGATTCTACCCCTCCTGCTGTGCCGACTAATCCAGTAGAAAAG
GGTTTGGCAGATATTTGGTCTCGTACGGCTGCACCTATGACAGCTAATGCCCGGACTGAATTTCGCCGTGCGATCCAGGATATGACTGATAGTTGGGTGTGGG
AACTGGCAAATCAGACCCAAAATCGGATTCCAGATCCGATAGATTATATTGAAATGCGTCGTAAGACGTTTGGCTCGGATTTGACGATGAGTCTGTCTCGATT
AGCCCAAGGTGGTGAGATTCCAATGGAAATCTACTACAGCCGACCAATGCGATCTCTGGAAAATTCCGCCGCAGACTTTGCCTGTTTCACCAATGACATTTTT
TCTTACCAGAAAGAAATCGAATTTGAAGGCGAAATTCATAACTGCGTATTGGTGGTTCAGAATTTCCTCAACTGCGATATAACCCAGGCTGTCGAGGTTGTTA
ACAATCTGATGACAGCGCGGGCGCAACAGTTTCAACACATCGTCGAGACTGAACTGCCAGCCCTTTTCGACGATTTCAAATTGGATGAAAACACCCGCGAGAA
ACTACTCAAATATGTTGAAAAACTAGAACAGTGGATGTGCGGTGTACTTAAGTGGCATATGAAGGTAGACCGCTATAAAGAATTTGAACTGCGTAATAGTGCT
TCGCCAATAGTGCGACTATTAAACGGTCCCACAGGGTTTGGCACTTCGGCTGCACATATCAGATCATTCGTCGGTGCAACCAATCTTTTTAGCCAAAATCTGT
CAACTTGA
