# Supplementary material for: Taxonomic and functional metagenomic assessment of a Dolichospermum bloom in a large and deep lake south of the Alps
Source: FEMS Microbiol Ecol. 2024 Sep 3;100(10):fiae117. doi: 10.1093/femsec/fiae117 (PMC11412076; doi:10.1093/femsec/fiae117)
Supplement: fiae117_Supplemental_Files [file fiae117_supplemental_files.zip › MAG_Dolichospermum_bloom_Suppl_Table_3.pdf]

Nico Salmaso, Leonardo Cerasino, Massimo Pindo, Adriano Boscaini - Taxonomic and functional metagenomic assessment of a *Dolichospermum* bloom in a large and deep lake south of the Alps

Supplementary Table 3

KEGG Mapper Reconstruction Result for the two NCBI *Dolichospermum lemmermannii* genomes. In the Pathway modules, functional units of genes are placed within the context of established metabolic pathways. A single M number or a combination of M numbers can be used for characterizing phenotypic features encoded in the genome (Kanehisa and Sato 2020). Examples are given in Supplementary Figures 4 and 5. Complete modules, modules including 1 block missing, and incomplete modules are reported in red-bold, red, and normal text, respectively. Blocks are essential steps within the metabolic pathways (Kanehisa, Sato and Kawashima 2022)

(A)

Dolichospermum lemmermannii FEM_B0920

Pathway modules

Carbohydrate metabolism

Central carbohydrate metabolism

M00001 Glycolysis (Embden-Meyerhof pathway), glucose => pyruvate (12) (complete 9/9)

M00002 Glycolysis, core module involving three-carbon compounds (7) (complete 5/5)

M00003 Gluconeogenesis, oxaloacetate => fructose-6P (9) (1 block missing 6/7)

M00307 Pyruvate oxidation, pyruvate => acetyl-CoA (5) (complete 1/1)

M00009 Citrate cycle (TCA cycle, Krebs cycle) (9) (incomplete 5/8)

M00010 Citrate cycle, first carbon oxidation, oxaloacetate => 2-oxoglutarate (3) (complete 3/3)

M00011 Citrate cycle, second carbon oxidation, 2-oxoglutarate => oxaloacetate (6) (incomplete 2/5)

M00004 Pentose phosphate pathway (Pentose phosphate cycle) (8) (complete 6/6)

M00006 Pentose phosphate pathway, oxidative phase, glucose 6P => ribulose 5P (3) (complete 2/2)

M00007 Pentose phosphate pathway, non-oxidative phase, fructose 6P => ribose 5P (4) (complete 4/4)

M00580 Pentose phosphate pathway, archaea, fructose 6P => ribose 5P (1) (1 block missing 1/2)

M00005 PRPP biosynthesis, ribose 5P => PRPP (1) (complete 1/1)

M00008 Entner-Doudoroff pathway, glucose-6P => glyceraldehyde-3P + pyruvate (3) (1 block missing 3/4)

M00308 Semi-phosphorylative Entner-Doudoroff pathway, gluconate => glycerate-3P (3) (2 blocks missing 2/4)

Other carbohydrate metabolism

M00014 Glucuronate pathway (uronate pathway) (2) (incomplete 2/7)

M00631 D-Galacturonate degradation (bacteria), D-galacturonate => pyruvate + D-glyceraldehyde 3P (1) (incomplete 1/5)

M00061 D-Glucuronate degradation, D-glucuronate => pyruvate + D-glyceraldehyde 3P (1) (incomplete 1/5)

M00632 Galactose degradation, Leloir pathway, galactose => alpha-D-glucose-1P (1) (incomplete 1/4)

M00552 D-galactonate degradation, De Ley-Doudoroff pathway, D-galactonate => glycerate-3P (2) (incomplete 2/5)

M00129 Ascorbate biosynthesis, animals, glucose-1P => ascorbate (1) (incomplete 1/7)

M00114 Ascorbate biosynthesis, plants, fructose-6P => ascorbate (2) (incomplete 2/8)

M00854 Glycogen biosynthesis, glucose-1P => glycogen/starch (4) (complete 2/2)

M00855 Glycogen degradation, glycogen => glucose-6P (2) (2 blocks missing 1/3)

M00565 Trehalose biosynthesis, D-glucose 1P => trehalose (4) (incomplete 3/6)

M00549 Nucleotide sugar biosynthesis, glucose => UDP-glucose (2) (2 blocks missing 1/3)

M00892 UDP-N-acetyl-D-glucosamine biosynthesis, eukaryotes, glucose => UDP-GlcNAc (3) (incomplete 3/6)

M00909 UDP-N-acetyl-D-glucosamine biosynthesis, prokaryotes, glucose => UDP-GlcNAc (6) (complete 5/5)

M00012 Glyoxylate cycle (2) (incomplete 2/5)

M00740 Methylaspartate cycle (3) (incomplete 3/11)

M00532 Photorespiration (9) (incomplete 5/10)

M00968 Pentose bisphosphate pathway (nucleoside degradation), archaea, nucleoside/NMP => 3-PGA/glycerone phosphate (1) (2 blocks missing 1/3)

M00131 Inositol phosphate metabolism, Ins(1,3,4,5)P4 => Ins(1,3,4)P3 => myo-inositol (1) (incomplete 1/4)

Energy metabolism

Carbon fixation

M00165 Reductive pentose phosphate cycle (Calvin cycle) (13) (complete 11/11)

(B)

Dolichospermum lemmermannii CS_548

Pathway modules

Carbohydrate metabolism

Central carbohydrate metabolism

M00001 Glycolysis (Embden-Meyerhof pathway), glucose => pyruvate (12) (complete 9/9)

M00002 Glycolysis, core module involving three-carbon compounds (7) (complete 5/5)

M00003 Gluconeogenesis, oxaloacetate => fructose-6P (9) (1 block missing 6/7)

M00307 Pyruvate oxidation, pyruvate => acetyl-CoA (5) (complete 1/1)

M00009 Citrate cycle (TCA cycle, Krebs cycle) (9) (incomplete 5/8)

M00010 Citrate cycle, first carbon oxidation, oxaloacetate => 2-oxoglutarate (3) (complete 3/3)

M00011 Citrate cycle, second carbon oxidation, 2-oxoglutarate => oxaloacetate (6) (incomplete 2/5)

M00004 Pentose phosphate pathway (Pentose phosphate cycle) (8) (complete 6/6)

M00006 Pentose phosphate pathway, oxidative phase, glucose 6P => ribulose 5P (3) (complete 2/2)

M00007 Pentose phosphate pathway, non-oxidative phase, fructose 6P => ribose 5P (4) (complete 4/4)

M00580 Pentose phosphate pathway, archaea, fructose 6P => ribose 5P (1) (1 block missing 1/2)

M00005 PRPP biosynthesis, ribose 5P => PRPP (1) (complete 1/1)

M00008 Entner-Doudoroff pathway, glucose-6P => glyceraldehyde-3P + pyruvate (3) (1 block missing 3/4)

M00308 Semi-phosphorylative Entner-Doudoroff pathway, gluconate => glycerate-3P (3) (2 blocks missing 2/4)

Other carbohydrate metabolism

M00014 Glucuronate pathway (uronate pathway) (2) (incomplete 2/7)

M00631 D-Galacturonate degradation (bacteria), D-galacturonate => pyruvate + D-glyceraldehyde 3P (1) (incomplete 1/5)

M00061 D-Glucuronate degradation, D-glucuronate => pyruvate + D-glyceraldehyde 3P (1) (incomplete 1/5)

M00632 Galactose degradation, Leloir pathway, galactose => alpha-D-glucose-1P (1) (incomplete 1/4)

M00552 D-galactonate degradation, De Ley-Doudoroff pathway, D-galactonate => glycerate-3P (2) (incomplete 2/5)

M00129 Ascorbate biosynthesis, animals, glucose-1P => ascorbate (1) (incomplete 1/7)

M00114 Ascorbate biosynthesis, plants, fructose-6P => ascorbate (2) (incomplete 2/8)

M00854 Glycogen biosynthesis, glucose-1P => glycogen/starch (4) (complete 2/2)

M00855 Glycogen degradation, glycogen => glucose-6P (1) (2 blocks missing 1/3)

M00565 Trehalose biosynthesis, D-glucose 1P => trehalose (4) (incomplete 3/6)

M00549 Nucleotide sugar biosynthesis, glucose => UDP-glucose (2) (2 blocks missing 1/3)

M00892 UDP-N-acetyl-D-glucosamine biosynthesis, eukaryotes, glucose => UDP-GlcNAc (3) (incomplete 3/6)

M00909 UDP-N-acetyl-D-glucosamine biosynthesis, prokaryotes, glucose => UDP-GlcNAc (6) (complete 5/5)

M00012 Glyoxylate cycle (2) (incomplete 2/5)

M00740 Methylaspartate cycle (3) (incomplete 3/11)

M00532 Photorespiration (9) (incomplete 5/10)

M00968 Pentose bisphosphate pathway (nucleoside degradation), archaea, nucleoside/NMP => 3-PGA/glycerone phosphate (1) (2 blocks missing 1/3)

M00131 Inositol phosphate metabolism, Ins(1,3,4,5)P4 => Ins(1,3,4)P3 => myo-inositol (1) (incomplete 1/4)

Energy metabolism

Carbon fixation

M00165 Reductive pentose phosphate cycle (Calvin cycle) (13) (complete 11/11)

M00168 CAM (Crassulacean acid metabolism), dark (1) (1 block missing 1/2)
M00172 C4-dicarboxylic acid cycle, NADP - malic enzyme type (1) (incomplete 1/4)
M00171 C4-dicarboxylic acid cycle, NAD - malic enzyme type (1) (incomplete 1/7)
M00170 C4-dicarboxylic acid cycle, phosphoenolpyruvate carboxykinase type (1) (incomplete 1/4)
M00173 Reductive citrate cycle (Arnon-Buchanan cycle) (10) (incomplete 6/10)
M00376 3-Hydroxypropionate bi-cycle (7) (incomplete 2/13)
M00374 Dicarboxylate-hydroxybutyrate cycle (7) (incomplete 4/13)
M00377 Reductive acetyl-CoA pathway (Wood-Ljungdahl pathway) (2) (incomplete 2/7)
M00579 Phosphate acetyltransferase-acetate kinase pathway, acetyl-CoA => acetate (1) (1 block missing 1/2)
M00620 Incomplete reductive citrate cycle, acetyl-CoA => oxoglutarate (2) (incomplete 1/7)

Methane metabolism

M00357 Methanogenesis, acetate => methane (2) (incomplete 1/5)
M00358 Coenzyme M biosynthesis (1) (incomplete 1/4)
M00346 Formaldehyde assimilation, serine pathway (4) (incomplete 4/9)
M00345 Formaldehyde assimilation, ribulose monophosphate pathway (2) (1 block missing 2/3)
M00344 Formaldehyde assimilation, xylulose monophosphate pathway (2) (2 blocks missing 2/4)
M00378 F420 biosynthesis, archaea (2) (incomplete 1/4)
M00935 Methanofuran biosynthesis (1) (incomplete 1/6)

Nitrogen metabolism

M00175 Nitrogen fixation, nitrogen => ammonia (3) (complete 1/1)
M00531 Assimilatory nitrate reduction, nitrate => ammonia (2) (complete 2/2)

Sulfur metabolism

M00176 Assimilatory sulfate reduction, sulfate => H2S (3) (1 block missing 1/2)
M00596 Dissimilatory sulfate reduction, sulfate => H2S (1) (incomplete 1/4)

Photosynthesis

M00161 Photosystem II (6) (complete 1/1)
M00163 Photosystem I (6) (complete 1/1)

ATP synthesis

M00145 NAD(P)H:quinone oxidoreductase, chloroplasts and cyanobacteria (13) (complete 1/1)
M00155 Cytochrome c oxidase, prokaryotes (3) (complete 1/1)
M00157 F-type ATPase, prokaryotes and chloroplasts (8) (complete 1/1)

Lipid metabolism

Fatty acid metabolism

M00082 Fatty acid biosynthesis, initiation (6) (complete 2/2)
M00083 Fatty acid biosynthesis, elongation (4) (complete 1/1)
M00873 Fatty acid biosynthesis in mitochondria, animals (2) (incomplete 1/6)
M00874 Fatty acid biosynthesis in mitochondria, fungi (3) (incomplete 2/6)
M00086 beta-Oxidation, acyl-CoA synthesis (1) (complete 1/1)

Sterol biosynthesis

M00101 Cholesterol biosynthesis, FPP => cholesterol (1) (incomplete 1/12)
M00102 Ergocalciferol biosynthesis, FPP => ergosterol/ergocalciferol (1) (incomplete 1/13)

Lipid metabolism

M00089 Triacylglycerol biosynthesis (2) (2 blocks missing 2/4)
M00098 Acylglycerol degradation (2) (complete 2/2)
M00093 Phosphatidylethanolamine (PE) biosynthesis, PA => PS => PE (1) (2 blocks missing 1/3)
M00066 Lactosylceramide biosynthesis (1) (1 block missing 1/2)

Nucleotide metabolism

Purine metabolism

M00048 De novo purine biosynthesis, PRPP + glutamine => IMP (13) (complete 8/8)
M00049 Adenine ribonucleotide biosynthesis, IMP => ADP,ATP (4) (complete 4/4)

M00168 CAM (Crassulacean acid metabolism), dark (1) (1 block missing 1/2)
M00172 C4-dicarboxylic acid cycle, NADP - malic enzyme type (1) (incomplete 1/4)
M00171 C4-dicarboxylic acid cycle, NAD - malic enzyme type (1) (incomplete 1/7)
M00170 C4-dicarboxylic acid cycle, phosphoenolpyruvate carboxykinase type (1) (incomplete 1/4)
M00173 Reductive citrate cycle (Arnon-Buchanan cycle) (10) (incomplete 6/10)
M00376 3-Hydroxypropionate bi-cycle (6) (incomplete 1/13)
M00374 Dicarboxylate-hydroxybutyrate cycle (7) (incomplete 4/13)
M00377 Reductive acetyl-CoA pathway (Wood-Ljungdahl pathway) (2) (incomplete 2/7)
M00579 Phosphate acetyltransferase-acetate kinase pathway, acetyl-CoA => acetate (1) (1 block missing 1/2)
M00620 Incomplete reductive citrate cycle, acetyl-CoA => oxoglutarate (2) (incomplete 1/7)

Methane metabolism

M00357 Methanogenesis, acetate => methane (2) (incomplete 1/5)
M00358 Coenzyme M biosynthesis (1) (incomplete 1/4)
M00346 Formaldehyde assimilation, serine pathway (4) (incomplete 4/9)
M00345 Formaldehyde assimilation, ribulose monophosphate pathway (2) (1 block missing 2/3)
M00344 Formaldehyde assimilation, xylulose monophosphate pathway (2) (2 blocks missing 2/4)
M00378 F420 biosynthesis, archaea (2) (incomplete 1/4)

Nitrogen metabolism

M00175 Nitrogen fixation, nitrogen => ammonia (3) (complete 1/1)
M00531 Assimilatory nitrate reduction, nitrate => ammonia (2) (complete 2/2)

Sulfur metabolism

M00176 Assimilatory sulfate reduction, sulfate => H2S (3) (1 block missing 1/2)
M00596 Dissimilatory sulfate reduction, sulfate => H2S (1) (incomplete 1/4)

Photosynthesis

M00161 Photosystem II (6) (complete 1/1)
M00163 Photosystem I (6) (complete 1/1)

ATP synthesis

M00145 NAD(P)H:quinone oxidoreductase, chloroplasts and cyanobacteria (13) (complete 1/1)
M00155 Cytochrome c oxidase, prokaryotes (3) (complete 1/1)
M00157 F-type ATPase, prokaryotes and chloroplasts (8) (complete 1/1)

Lipid metabolism

Fatty acid metabolism

M00082 Fatty acid biosynthesis, initiation (5) (1 block missing 1/2)
M00083 Fatty acid biosynthesis, elongation (4) (complete 1/1)
M00873 Fatty acid biosynthesis in mitochondria, animals (2) (incomplete 1/6)
M00874 Fatty acid biosynthesis in mitochondria, fungi (3) (incomplete 2/6)
M00086 beta-Oxidation, acyl-CoA synthesis (1) (complete 1/1)

Sterol biosynthesis

M00101 Cholesterol biosynthesis, FPP => cholesterol (1) (incomplete 1/12)
M00102 Ergocalciferol biosynthesis, FPP => ergosterol/ergocalciferol (1) (incomplete 1/13)

Lipid metabolism

M00089 Triacylglycerol biosynthesis (2) (2 blocks missing 2/4)
M00098 Acylglycerol degradation (2) (complete 2/2)
M00093 Phosphatidylethanolamine (PE) biosynthesis, PA => PS => PE (1) (2 blocks missing 1/3)
M00066 Lactosylceramide biosynthesis (1) (1 block missing 1/2)

Nucleotide metabolism

Purine metabolism

M00048 De novo purine biosynthesis, PRPP + glutamine => IMP (12) (complete 8/8)
M00049 Adenine ribonucleotide biosynthesis, IMP => ADP,ATP (4) (complete 4/4)

M00050 Guanine ribonucleotide biosynthesis, IMP => GDP,GTP (4) (complete 4/4)
M00053 Deoxyribonucleotide biosynthesis, ADP/GDP/CDP/UDP => dATP/dGTP/dCTP/dUTP (1) (1 block missing 1/2)
M00958 Adenine ribonucleotide degradation, AMP => Urate (3) (1 block missing 2/3)
M00959 Guanine ribonucleotide degradation, GMP => Urate (2) (2 blocks missing 2/4)

Pyrimidine metabolism

M00051 De novo pyrimidine biosynthesis, glutamine (+ PRPP) => UMP (9) (1 block missing 2/3)
M00052 Pyrimidine ribonucleotide biosynthesis, UMP => UDP/UTP,CDP/CTP (3) (complete 3/3)
M00938 Pyrimidine deoxyribonucleotide biosynthesis, UDP => dTTP (4) (1 block missing 4/5)
M00939 Pyrimidine degradation, uracil => 3-hydroxypropanoate (1) (incomplete 1/5)

Amino acid metabolism

Serine and threonine metabolism

M00020 Serine biosynthesis, glycerate-3P => serine (1) (2 blocks missing 1/3)
M00018 Threonine biosynthesis, aspartate => homoserine => threonine (5) (complete 5/5)
M00621 Glycine cleavage system (3) (complete 3/3)
M00975 Betaine degradation, bacteria, betaine => pyruvate (2) (2 blocks missing 2/4)
M00033 Ectoine biosynthesis, aspartate => ectoine (2) (incomplete 2/5)

Cysteine and methionine metabolism

M00021 Cysteine biosynthesis, serine => cysteine (2) (complete 2/2)
M00609 Cysteine biosynthesis, methionine => cysteine (1) (incomplete 1/6)
M00017 Methionine biosynthesis, aspartate => homoserine => methionine (5) (2 blocks missing 5/7)
M00034 Methionine salvage pathway (4) (incomplete 3/8)
M00035 Methionine degradation (3) (1 block missing 3/4)
M00368 Ethylene biosynthesis, methionine => ethylene (1) (2 blocks missing 1/3)

Branched-chain amino acid metabolism

M00019 Valine/isoleucine biosynthesis, pyruvate => valine / 2-oxobutanoate => isoleucine (5) (complete 4/4)
M00535 Isoleucine biosynthesis, pyruvate => 2-oxobutanoate (3) (1 block missing 2/3)
M00570 Isoleucine biosynthesis, threonine => 2-oxobutanoate => isoleucine (6) (complete 5/5)
M00432 Leucine biosynthesis, 2-oxoisovalerate => 2-oxoisocaproate (4) (complete 3/3)
M00036 Leucine degradation, leucine => acetoacetate + acetyl-CoA (2) (incomplete 1/6)

Lysine metabolism

M00016 Lysine biosynthesis, succinyl-DAP pathway, aspartate => lysine (7) (2 blocks missing 7/9)
M00525 Lysine biosynthesis, acetyl-DAP pathway, aspartate => lysine (6) (incomplete 6/9)
M00526 Lysine biosynthesis, DAP dehydrogenase pathway, aspartate => lysine (5) (1 block missing 5/6)
M00527 Lysine biosynthesis, DAP aminotransferase pathway, aspartate => lysine (7) (complete 7/7)
M00956 Lysine degradation, bacteria, L-lysine => succinate (2) (incomplete 2/7)
M00957 Lysine degradation, bacteria, L-lysine => glutarate => succinate/acetyl-CoA (2) (incomplete 1/5)

Arginine and proline metabolism

M00028 Ornithine biosynthesis, glutamate => ornithine (4) (complete 4/4)
M00844 Arginine biosynthesis, ornithine => arginine (3) (complete 3/3)
M00845 Arginine biosynthesis, glutamate => acetylcitrulline => arginine (4) (incomplete 4/7)
M00029 Urea cycle (3) (2 blocks missing 3/5)
M00015 Proline biosynthesis, glutamate => proline (3) (complete 2/2)
M00970 Proline degradation, proline => glutamate (1) (complete 1/1)
M00972 Proline metabolism (1) (2 blocks missing 1/3)

Polyamine biosynthesis

M00133 Polyamine biosynthesis, arginine => agmatine => putrescine => spermidine (2) (2 blocks missing 2/4)
M00135 GABA biosynthesis, eukaryotes, putrescine => GABA (1) (2 blocks missing 1/3)

Histidine metabolism

M00026 Histidine biosynthesis, PRPP => histidine (10) (1 block missing 5/6)

Aromatic amino acid metabolism

M00022 Shikimate pathway, phosphoenolpyruvate + erythrose-4P => chorismate (7) (1 block missing 3/4)

M00050 Guanine ribonucleotide biosynthesis, IMP => GDP,GTP (4) (complete 4/4)
M00053 Deoxyribonucleotide biosynthesis, ADP/GDP/CDP/UDP => dATP/dGTP/dCTP/dUTP (1) (1 block missing 1/2)
M00958 Adenine ribonucleotide degradation, AMP => Urate (3) (1 block missing 2/3)
M00959 Guanine ribonucleotide degradation, GMP => Urate (2) (2 blocks missing 2/4)

Pyrimidine metabolism

M00051 De novo pyrimidine biosynthesis, glutamine (+ PRPP) => UMP (8) (1 block missing 2/3)
M00052 Pyrimidine ribonucleotide biosynthesis, UMP => UDP/UTP,CDP/CTP (3) (complete 3/3)
M00938 Pyrimidine deoxyribonucleotide biosynthesis, UDP => dTTP (3) (2 blocks missing 3/5)
M00939 Pyrimidine degradation, uracil => 3-hydroxypropanoate (1) (incomplete 1/5)

Amino acid metabolism

Serine and threonine metabolism

M00020 Serine biosynthesis, glycerate-3P => serine (1) (2 blocks missing 1/3)
M00018 Threonine biosynthesis, aspartate => homoserine => threonine (5) (complete 5/5)
M00621 Glycine cleavage system (3) (complete 3/3)
M00975 Betaine degradation, bacteria, betaine => pyruvate (2) (2 blocks missing 2/4)
M00033 Ectoine biosynthesis, aspartate => ectoine (2) (incomplete 2/5)

Cysteine and methionine metabolism

M00021 Cysteine biosynthesis, serine => cysteine (2) (complete 2/2)
M00609 Cysteine biosynthesis, methionine => cysteine (1) (incomplete 1/6)
M00017 Methionine biosynthesis, aspartate => homoserine => methionine (5) (2 blocks missing 5/7)
M00034 Methionine salvage pathway (4) (incomplete 3/8)
M00035 Methionine degradation (3) (1 block missing 3/4)
M00368 Ethylene biosynthesis, methionine => ethylene (1) (2 blocks missing 1/3)

Branched-chain amino acid metabolism

M00019 Valine/isoleucine biosynthesis, pyruvate => valine / 2-oxobutanoate => isoleucine (5) (complete 4/4)
M00535 Isoleucine biosynthesis, pyruvate => 2-oxobutanoate (3) (1 block missing 2/3)
M00570 Isoleucine biosynthesis, threonine => 2-oxobutanoate => isoleucine (6) (complete 5/5)
M00432 Leucine biosynthesis, 2-oxoisovalerate => 2-oxoisocaproate (4) (complete 3/3)
M00036 Leucine degradation, leucine => acetoacetate + acetyl-CoA (2) (incomplete 1/6)

Lysine metabolism

M00016 Lysine biosynthesis, succinyl-DAP pathway, aspartate => lysine (7) (2 blocks missing 7/9)
M00525 Lysine biosynthesis, acetyl-DAP pathway, aspartate => lysine (6) (incomplete 6/9)
M00526 Lysine biosynthesis, DAP dehydrogenase pathway, aspartate => lysine (5) (1 block missing 5/6)
M00527 Lysine biosynthesis, DAP aminotransferase pathway, aspartate => lysine (7) (complete 7/7)
M00956 Lysine degradation, bacteria, L-lysine => succinate (2) (incomplete 2/7)
M00957 Lysine degradation, bacteria, L-lysine => glutarate => succinate/acetyl-CoA (2) (incomplete 1/5)

Arginine and proline metabolism

M00028 Ornithine biosynthesis, glutamate => ornithine (4) (complete 4/4)
M00844 Arginine biosynthesis, ornithine => arginine (3) (complete 3/3)
M00845 Arginine biosynthesis, glutamate => acetylcitrulline => arginine (4) (incomplete 4/7)
M00029 Urea cycle (3) (2 blocks missing 3/5)
M00015 Proline biosynthesis, glutamate => proline (3) (complete 2/2)
M00970 Proline degradation, proline => glutamate (1) (complete 1/1)
M00972 Proline metabolism (1) (2 blocks missing 1/3)

Polyamine biosynthesis

M00133 Polyamine biosynthesis, arginine => agmatine => putrescine => spermidine (2) (2 blocks missing 2/4)
M00135 GABA biosynthesis, eukaryotes, putrescine => GABA (1) (2 blocks missing 1/3)

Histidine metabolism

M00026 Histidine biosynthesis, PRPP => histidine (9) (1 block missing 5/6)

Aromatic amino acid metabolism

M00022 Shikimate pathway, phosphoenolpyruvate + erythrose-4P => chorismate (7) (1 block missing 3/4)

M00023 Tryptophan biosynthesis, chorismate => tryptophan (7) (complete 3/3)
M00024 Phenylalanine biosynthesis, chorismate => phenylpyruvate => phenylalanine (2) (1 block missing 1/2)
M00044 Tyrosine degradation, tyrosine => homogentisate (1) (incomplete 1/5)

Other amino acid metabolism
M00027 GABA (gamma-Aminobutyrate) shunt (1) (2 blocks missing 1/3)
M00118 Glutathione biosynthesis, glutamate => glutathione (1) (1 block missing 1/2)

Glycan metabolism

Glycan biosynthesis
M00872 O-glycan biosynthesis, mannose type (core M3) (1) (incomplete 1/10)

Lipopolysaccharide metabolism
M00060 KDO2-lipid A biosynthesis, Raetz pathway, LpxL-LpxM type (4) (incomplete 4/9)
M00866 KDO2-lipid A biosynthesis, Raetz pathway, non-LpxL-LpxM type (4) (incomplete 4/9)

Metabolism of cofactors and vitamins

Cofactor and vitamin metabolism
M00127 Thiamine biosynthesis, prokaryotes, AIR (+ DXP/tyrosine) => TMP/TPP (6) (incomplete 4/7)
M00895 Thiamine biosynthesis, prokaryotes, AIR (+ DXP/glycine) => TMP/TPP (6) (incomplete 5/9)
M00896 Thiamine biosynthesis, archaea, AIR (+ NAD+) => TMP/TPP (4) (1 block missing 3/4)
M00897 Thiamine biosynthesis, plants, AIR (+ NAD+) => TMP/thiamine/TPP (1) (incomplete 1/5)
M00899 Thiamine salvage pathway, HMP/HET => TMP (2) (1 block missing 1/2)
M00125 Riboflavin biosynthesis, plants and bacteria, GTP => riboflavin/FMN/FAD (5) (1 block missing 6/7)
M00911 Riboflavin biosynthesis, fungi, GTP => riboflavin/FMN/FAD (2) (incomplete 2/8)
M00124 Pyridoxal-P biosynthesis, erythrose-4P => pyridoxal-P (2) (incomplete 2/6)
M00115 NAD biosynthesis, aspartate => quinolinate => NAD (5) (complete 5/5)
M00912 NAD biosynthesis, tryptophan => quinolinate => NAD (3) (incomplete 3/8)
M00119 Pantothenate biosynthesis, valine/L-aspartate => pantothenate (4) (1 block missing 4/5)
M00913 Pantothenate biosynthesis, 2-oxoisovalerate/spermine => pantothenate (2) (incomplete 2/5)
M00120 Coenzyme A biosynthesis, pantothenate => CoA (4) (complete 3/3)
M00914 Coenzyme A biosynthesis, archaea, 2-oxoisovalerate => 4-phosphopantoate => CoA (2) (incomplete 2/7)
M00572 Pimeloyl-ACP biosynthesis, BioC-BioH pathway, malonyl-ACP => pimeloyl-ACP (4) (2 blocks missing 4/6)
M00123 Biotin biosynthesis, pimeloyl-ACP/CoA => biotin (3) (1 block missing 2/3)
M00950 Biotin biosynthesis, BioU pathway, pimeloyl-ACP/CoA => biotin (4) (complete 4/4)
M00573 Biotin biosynthesis, BioI pathway, long-chain-acyl-ACP => pimeloyl-ACP => biotin (3) (2 blocks missing 3/5)
M00577 Biotin biosynthesis, BioW pathway, pimelate => pimeloyl-CoA => biotin (3) (2 blocks missing 3/5)
M00881 Lipoic acid biosynthesis, plants and bacteria, octanoyl-ACP => dihydrolipoyl-E2/H (2) (complete 2/2)
M00882 Lipoic acid biosynthesis, eukaryotes, octanoyl-ACP => dihydrolipoyl-H (1) (1 block missing 1/2)
M00883 Lipoic acid biosynthesis, animals and bacteria, octanoyl-ACP => dihydrolipoyl-H => dihydrolipoyl-E2 (1) (2 blocks missing 1/3)
M00884 Lipoic acid biosynthesis, octanoyl-CoA => dihydrolipoyl-E2 (1) (1 block missing 1/2)
M00126 Tetrahydrofolate biosynthesis, GTP => THF (7) (complete 5/5)
M00840 Tetrahydrofolate biosynthesis, mediated by ribA and trpF, GTP => THF (3) (incomplete 3/6)
M00841 Tetrahydrofolate biosynthesis, mediated by PTPS, GTP => THF (2) (incomplete 2/5)
M00842 Tetrahydrobiopterin biosynthesis, GTP => BH4 (2) (1 block missing 2/3)
M00843 L-threo-Tetrahydrobiopterin biosynthesis, GTP => L-threo-BH4 (2) (1 block missing 2/3)
M00880 Molybdenum cofactor biosynthesis, GTP => molybdenum cofactor (5) (complete 3/3)
M00140 C1-unit interconversion, prokaryotes (2) (1 block missing 2/3)
M00141 C1-unit interconversion, eukaryotes (1) (1 block missing 1/2)
M00846 Siroheme biosynthesis, glutamyl-tRNA => siroheme (6) (1 block missing 5/6)
M00868 Heme biosynthesis, animals and fungi, glycine => heme (5) (incomplete 5/8)
M00121 Heme biosynthesis, plants and bacteria, glutamate => heme (11) (complete 10/10)
M00926 Heme biosynthesis, bacteria, glutamyl-tRNA => coproporphyrin III => heme (7) (2 blocks missing 7/9)
M00924 Cobalamin biosynthesis, anaerobic, uroporphyrinogen III => sirohydrochlorin => cobyrinate a,c-diamide (12) (1 block missing 10/11)
M00925 Cobalamin biosynthesis, aerobic, uroporphyrinogen III => precorrin 2 => cobyrinate a,c-diamide (10) (incomplete 8/11)
M00122 Cobalamin biosynthesis, cobyrinate a,c-diamide => cobalamin (6) (2 blocks missing 5/7)
M00117 Ubiquinone biosynthesis, prokaryotes, chorismate (+ polyprenyl-PP) => ubiquinol (5) (incomplete 4/9)
M00116 Menaquinone biosynthesis, chorismate (+ polyprenyl-PP) => menaquinol (6) (incomplete 6/9)

M00023 Tryptophan biosynthesis, chorismate => tryptophan (7) (complete 3/3)
M00024 Phenylalanine biosynthesis, chorismate => phenylpyruvate => phenylalanine (2) (1 block missing 1/2)
M00044 Tyrosine degradation, tyrosine => homogentisate (1) (incomplete 1/5)

Other amino acid metabolism
M00027 GABA (gamma-Aminobutyrate) shunt (1) (2 blocks missing 1/3)
M00118 Glutathione biosynthesis, glutamate => glutathione (1) (1 block missing 1/2)

Glycan metabolism

Glycan biosynthesis
M00872 O-glycan biosynthesis, mannose type (core M3) (1) (incomplete 1/10)

Lipopolysaccharide metabolism
M00060 KDO2-lipid A biosynthesis, Raetz pathway, LpxL-LpxM type (4) (incomplete 4/9)
M00866 KDO2-lipid A biosynthesis, Raetz pathway, non-LpxL-LpxM type (4) (incomplete 4/9)
M00064 ADP-L-glycero-D-manno-heptose biosynthesis (2) (incomplete 2/5)

Metabolism of cofactors and vitamins

Cofactor and vitamin metabolism
M00127 Thiamine biosynthesis, prokaryotes, AIR (+ DXP/tyrosine) => TMP/TPP (6) (incomplete 4/7)
M00895 Thiamine biosynthesis, prokaryotes, AIR (+ DXP/glycine) => TMP/TPP (6) (incomplete 5/9)
M00896 Thiamine biosynthesis, archaea, AIR (+ NAD+) => TMP/TPP (4) (1 block missing 3/4)
M00897 Thiamine biosynthesis, plants, AIR (+ NAD+) => TMP/thiamine/TPP (1) (incomplete 1/5)
M00899 Thiamine salvage pathway, HMP/HET => TMP (2) (1 block missing 1/2)
M00125 Riboflavin biosynthesis, plants and bacteria, GTP => riboflavin/FMN/FAD (4) (2 blocks missing 5/7)
M00911 Riboflavin biosynthesis, fungi, GTP => riboflavin/FMN/FAD (1) (incomplete 1/8)
M00124 Pyridoxal-P biosynthesis, erythrose-4P => pyridoxal-P (2) (incomplete 2/6)
M00115 NAD biosynthesis, aspartate => quinolinate => NAD (5) (complete 5/5)
M00912 NAD biosynthesis, tryptophan => quinolinate => NAD (3) (incomplete 3/8)
M00119 Pantothenate biosynthesis, valine/L-aspartate => pantothenate (4) (1 block missing 4/5)
M00913 Pantothenate biosynthesis, 2-oxoisovalerate/spermine => pantothenate (2) (incomplete 2/5)
M00120 Coenzyme A biosynthesis, pantothenate => CoA (3) (1 block missing 2/3)
M00914 Coenzyme A biosynthesis, archaea, 2-oxoisovalerate => 4-phosphopantoate => CoA (2) (incomplete 2/7)
M00572 Pimeloyl-ACP biosynthesis, BioC-BioH pathway, malonyl-ACP => pimeloyl-ACP (4) (2 blocks missing 4/6)
M00123 Biotin biosynthesis, pimeloyl-ACP/CoA => biotin (3) (1 block missing 2/3)
M00950 Biotin biosynthesis, BioU pathway, pimeloyl-ACP/CoA => biotin (4) (complete 4/4)
M00573 Biotin biosynthesis, BioI pathway, long-chain-acyl-ACP => pimeloyl-ACP => biotin (3) (2 blocks missing 3/5)
M00577 Biotin biosynthesis, BioW pathway, pimelate => pimeloyl-CoA => biotin (3) (2 blocks missing 3/5)
M00881 Lipoic acid biosynthesis, plants and bacteria, octanoyl-ACP => dihydrolipoyl-E2/H (2) (complete 2/2)
M00882 Lipoic acid biosynthesis, eukaryotes, octanoyl-ACP => dihydrolipoyl-H (1) (1 block missing 1/2)
M00883 Lipoic acid biosynthesis, animals and bacteria, octanoyl-ACP => dihydrolipoyl-H => dihydrolipoyl-E2 (1) (2 blocks missing 1/3)
M00884 Lipoic acid biosynthesis, octanoyl-CoA => dihydrolipoyl-E2 (1) (1 block missing 1/2)
M00126 Tetrahydrofolate biosynthesis, GTP => THF (7) (complete 5/5)
M00840 Tetrahydrofolate biosynthesis, mediated by ribA and trpF, GTP => THF (3) (incomplete 3/6)
M00841 Tetrahydrofolate biosynthesis, mediated by PTPS, GTP => THF (2) (incomplete 2/5)
M00842 Tetrahydrobiopterin biosynthesis, GTP => BH4 (2) (1 block missing 2/3)
M00843 L-threo-Tetrahydrobiopterin biosynthesis, GTP => L-threo-BH4 (2) (1 block missing 2/3)
M00880 Molybdenum cofactor biosynthesis, GTP => molybdenum cofactor (5) (complete 3/3)
M00140 C1-unit interconversion, prokaryotes (2) (1 block missing 2/3)
M00141 C1-unit interconversion, eukaryotes (1) (1 block missing 1/2)
M00846 Siroheme biosynthesis, glutamyl-tRNA => siroheme (6) (1 block missing 5/6)
M00868 Heme biosynthesis, animals and fungi, glycine => heme (5) (incomplete 5/8)
M00121 Heme biosynthesis, plants and bacteria, glutamate => heme (11) (complete 10/10)
M00926 Heme biosynthesis, bacteria, glutamyl-tRNA => coproporphyrin III => heme (7) (2 blocks missing 7/9)
M00924 Cobalamin biosynthesis, anaerobic, uroporphyrinogen III => sirohydrochlorin => cobyrinate a,c-diamide (12) (1 block missing 10/11)
M00925 Cobalamin biosynthesis, aerobic, uroporphyrinogen III => precorrin 2 => cobyrinate a,c-diamide (10) (incomplete 8/11)
M00122 Cobalamin biosynthesis, cobyrinate a,c-diamide => cobalamin (6) (2 blocks missing 5/7)
M00117 Ubiquinone biosynthesis, prokaryotes, chorismate (+ polyprenyl-PP) => ubiquinol (5) (incomplete 4/9)
M00116 Menaquinone biosynthesis, chorismate (+ polyprenyl-PP) => menaquinol (6) (incomplete 6/9)

M00932 Phylloquinone biosynthesis, chorismate (+ phytyl-PP) => phylloquinol (10) (complete 7/7)
M00112 Tocopherol/tocotorienol biosynthesis, homogentisate + phytyl/geranylgeranyl-PP => tocopherol/tocotorienol (3) (1 block missing 3/4)

Biosynthesis of terpenoids and polyketides

Terpenoid backbone biosynthesis
M00095 C5 isoprenoid biosynthesis, mevalonate pathway (1) (incomplete 1/7)
M00849 C5 isoprenoid biosynthesis, mevalonate pathway, archaea (2) (incomplete 1/6)
M00096 C5 isoprenoid biosynthesis, non-mevalonate pathway (8) (complete 8/8)
M00364 C10-C20 isoprenoid biosynthesis, bacteria (2) (complete 2/2)
M00365 C10-C20 isoprenoid biosynthesis, archaea (1) (1 block missing 1/2)
M00366 C10-C20 isoprenoid biosynthesis, plants (2) (2 blocks missing 2/4)
M00367 C10-C20 isoprenoid biosynthesis, non-plant eukaryotes (1) (2 blocks missing 1/3)

Plant terpenoid biosynthesis
M00097 beta-Carotene biosynthesis, GGAP => beta-carotene (4) (2 blocks missing 4/6)

Polyketide sugar unit biosynthesis
M00793 dTDP-L-rhamnose biosynthesis (4) (complete 3/3)

Biosynthesis of other secondary metabolites

Biosynthesis of phytochemical compounds
M00953 Mugineic acid biosynthesis, methionine => 3-epihydroxymugineic acid (1) (incomplete 1/5)

Biosynthesis of other antibiotics

M00932 Phylloquinone biosynthesis, chorismate (+ phytyl-PP) => phylloquinol (10) (complete 7/7)
M00112 Tocopherol/tocotorienol biosynthesis, homogentisate + phytyl/geranylgeranyl-PP => tocopherol/tocotorienol (3) (1 block missing 3/4)

Biosynthesis of terpenoids and polyketides

Terpenoid backbone biosynthesis
M00095 C5 isoprenoid biosynthesis, mevalonate pathway (1) (incomplete 1/7)
M00849 C5 isoprenoid biosynthesis, mevalonate pathway, archaea (2) (incomplete 1/6)
M00096 C5 isoprenoid biosynthesis, non-mevalonate pathway (8) (complete 8/8)
M00364 C10-C20 isoprenoid biosynthesis, bacteria (2) (complete 2/2)
M00365 C10-C20 isoprenoid biosynthesis, archaea (1) (1 block missing 1/2)
M00366 C10-C20 isoprenoid biosynthesis, plants (2) (2 blocks missing 2/4)
M00367 C10-C20 isoprenoid biosynthesis, non-plant eukaryotes (1) (2 blocks missing 1/3)

Plant terpenoid biosynthesis
M00097 beta-Carotene biosynthesis, GGAP => beta-carotene (5) (1 block missing 5/6)

Polyketide sugar unit biosynthesis
M00793 dTDP-L-rhamnose biosynthesis (4) (complete 3/3)

Biosynthesis of other secondary metabolites

Biosynthesis of phytochemical compounds
M00953 Mugineic acid biosynthesis, methionine => 3-epihydroxymugineic acid (1) (incomplete 1/5)

Biosynthesis of other antibiotics
M00877 Kanosamine biosynthesis, glucose 6-phosphate => kanosamine (1) (2 blocks missing 1/3)
