# Supplementary material for: Taxonomic and functional metagenomic assessment of a Dolichospermum bloom in a large and deep lake south of the Alps
Source: FEMS Microbiol Ecol. 2024 Sep 3;100(10):fiae117. doi: 10.1093/femsec/fiae117 (PMC11412076; doi:10.1093/femsec/fiae117)
Supplement: fiae117_Supplemental_Files [file fiae117_supplemental_files.zip › MAG_Dolichospermum_bloom_Suppl_Table_4.pdf]

Taxonomic and functional metagenomic assessment of a *Dolichospermum* bloom in a large and deep lake south of the Alps

Nico Salmaso, Leonardo Cerasino, Massimo Pindo, Adriano Boscaini

Supplementary Table 4

Supplementary Table 4. Bacterial MAGs identified after the binning steps. Classification was performed using gtdb-tk 2.4.0 updated to use the GTDB R220 taxonomy; Class_type, ANI and placement-tree: classification of the query genomes based on either ANI or their placement in the reference tree (Chaumeil et al., 2022). Compl., completeness and Cont., contamination estimated by CheckM 2 (Chklovski *et al.* 2023). Size, genome size (bp); GC, GC content. Mean_Cov, mean coverage and Rel_ab, relative abundance (%) estimated by CoverM 0.6.1 (github.com/wwood/CoverM); unmapped reads were not reported in the table. Classes are highlighted in different colors. All reported taxa are bacteria (d__Bacteria); other codes: p__ Phylum; c__ Class; o__ Order; f__ Family; g__ Genus; s__ Species.

Classification	Class_type	Compl.	Cont.	Size	GC	Mean_cov	Rel_ab
p__Cyanobacteriota;c__Cyanobacteriia;o__Cyanobacteriales; f__Nostocaceae;g__*Dolichospermum*;s__*Dolichospermum sp000312705*	ANI	99.9	0.0	4,787,045	0.38	305.3	26.3
p__Myxococcota;c__Polyangia;o__Polyangiales; f__Polyangiaceae;g__JAAFHV01;s__	placement-tree	67.7	3.5	6,351,217	0.66	5.3	0.5
p__Planctomycetota;c__Phycisphaerae;o__Phycisphaerales; f__UBA1924;g__JAJTHN01;s__JAJTHN01 sp021297895	ANI	45.8	2.0	2,321,844	0.67	5.3	0.5
p__Pseudomonadota;c__Alphaproteobacteria;o__CACIAM-22H2; f__CACIAM-22H2;g__*Tagaea*;s__*Tagaea sp027532905*	ANI	99.0	5.1	4,418,608	0.64	41.8	3.6
p__Pseudomonadota;c__Alphaproteobacteria;o__CACIAM-22H2; f__CACIAM-22H2;g__*Tagaea*;s__*Tagaea sp945901855*	ANI	96.6	0.4	3,680,316	0.64	15.9	1.4
p__Pseudomonadota;c__Alphaproteobacteria;o__Elsterales; f__Elsteraceae;g__;s__	placement-tree	76.2	6.0	4,010,084	0.66	8.2	0.7
p__Pseudomonadota;c__Alphaproteobacteria;o__Rhizobiales; f__Beijerinckiaceae;g__*Rhabdaerophilum*;s__*Rhabdaerophilum sp027484605*	ANI	98.9	0.1	4,057,205	0.65	46.3	4.0
p__Pseudomonadota;c__Alphaproteobacteria;o__Rhizobiales; f__Beijerinckiaceae;g__*Rhabdaerophilum*;s__	placement-tree	75.6	1.7	3,116,997	0.62	6.6	0.6
p__Pseudomonadota;c__Alphaproteobacteria;o__Sphingomonadales; f__Sphingomonadaceae;g__*Sphingorhabdus*_B;s__*Sphingorhabdus_B sp016462305*	ANI	100.0	0.3	2,687,785	0.55	323.4	27.8
p__Pseudomonadota;c__Gammaproteobacteria;o__Burkholderiales; f__Burkholderiaceae;g__*Acidovorax*;s__*Acidovorax sp001464865*	ANI	85.2	6.7	4,368,772	0.66	9.9	0.9
p__Pseudomonadota;c__Gammaproteobacteria;o__Burkholderiales; f__Burkholderiaceae;g__*Acidovorax*;s__*Acidovorax sp003060895*	ANI	95.6	5.8	4,809,422	0.65	14.2	1.2
p__Pseudomonadota;c__Gammaproteobacteria;o__Burkholderiales; f__Burkholderiaceae;g__*Rubrivivax*;s__	placement-tree	99.7	0.6	5,314,297	0.7	35.7	3.1
p__Pseudomonadota;c__Gammaproteobacteria;o__Burkholderiales; f__Burkholderiaceae;g__*Rubrivivax*;s__	placement-tree	100.0	3.8	5,267,354	0.67	14.2	1.2
p__Pseudomonadota;c__Gammaproteobacteria;o__Burkholderiales; f__Burkholderiaceae;g__*JAIXUC01*;s__	placement-tree	84.2	22.4	4,916,355	0.63	5.1	0.4
p__Pseudomonadota;c__Gammaproteobacteria;o__Xanthomonadales; f__Xanthomonadaceae;g__*SCMT01*;s__	placement-tree	100.0	0.5	4,551,648	0.63	146.5	12.6
